# Therapist-Guided Internet-Delivered Cognitive Behavioral Therapy vs Internet-Delivered Supportive Therapy for Children and Adolescents With Social Anxiety Disorder: A Randomized Clinical Trial

**DOI:** 10.1001/jamapsychiatry.2021.0469

**Published:** 2021-05-12

**Authors:** Martina Nordh, Tove Wahlund, Maral Jolstedt, Hanna Sahlin, Johan Bjureberg, Johan Ahlen, Maria Lalouni, Sigrid Salomonsson, Sarah Vigerland, Malin Lavner, Lars-Göran Öst, Fabian Lenhard, Hugo Hesser, David Mataix-Cols, Jens Högström, Eva Serlachius

**Affiliations:** 1 Centre for Psychiatry Research, Department of Clinical Neuroscience, Karolinska Institutet and Stockholm Health Care Services, Region Stockholm, Stockholm, Sweden; 2 Department of Global Public Health, Karolinska Institutet, Stockholm, Sweden; 3 Division of Neuro, Department of Clinical Neuroscience, Karolinska Institutet, Stockholm, Sweden; 4 Department of Psychology, Stockholm University, Stockholm, Sweden; 5 Center for Health and Medical Psychology, Örebro University, Örebro, Sweden; 6 Department of Behavioral Sciences and Learning, Linköping University, Linköping, Sweden

## Abstract

**Importance:**

Social anxiety disorder (SAD) is a prevalent childhood-onset disorder associated with lifelong adversity and high costs for the individual and society at large. Cognitive behavioral therapy (CBT) is an established evidence-based treatment for SAD, but its availability is limited.

**Objective:**

To assess the efficacy and cost-effectiveness of therapist-guided internet-delivered cognitive behavioral therapy (ICBT) for SAD in youths vs an active comparator, internet-delivered supportive therapy (ISUPPORT).

**Design, Setting, and Participants:**

This single-masked, superiority randomized clinical trial enrolled participants at a clinical research unit integrated within the child and adolescent mental health services in Stockholm, Sweden, from September 1, 2017, to October 31, 2018. The final participant reached the 3-month follow-up (primary end point) in May 2019. Children and adolescents 10 to 17 years of age with a principal diagnosis of SAD and their parents were included in the study.

**Interventions:**

ICBT and ISUPPORT, both including 10 online modules, 5 separate parental modules, and 3 video call sessions with a therapist.

**Main Outcomes and Measures:**

The Clinician Severity Rating (CSR), derived from the Anxiety Disorder Interview Schedule, rated by masked assessors 3 months after the end of treatment. The CSR ranges from 0 to 8, with scores of 4 or higher indicating caseness. Secondary outcomes included masked assessor–rated diagnostic status of SAD and global functioning, child- and parent-reported social anxiety and depressive symptoms, and health-related costs.

**Results:**

Of the 307 youths assessed for eligibility, 103 were randomized to 10 weeks of therapist-guided ICBT (n = 51) or therapist-guided ISUPPORT (n = 52) for SAD. The sample consisted of 103 youths (mean [SD] age, 14.1 [2.1] years; 79 [77%] female). Internet-delivered cognitive behavioral therapy was significantly more efficacious than ISUPPORT in reducing the severity of SAD symptoms. Mean (SD) CSR scores for ICBT at baseline and at the 3-month follow-up were 5.06 (0.95) and 3.96 (1.46), respectively, compared with 4.94 (0.94) and 4.48 (1.30) for ISUPPORT. There was a significant between-group effect size of *d* = 0.67 (95% CI, 0.21-1.12) at the 3-month follow-up. Similarly, all of the secondary outcome measures demonstrated significant differences with small to large effect sizes, except for child-rated quality of life (nonsignificant). The cost-effectiveness analyses indicated cost savings associated with ICBT compared with ISUPPORT, with the main drivers of the savings being lower medication costs (*z* = 2.38, *P* = .02) and increased school productivity (*z* = 1.99, *P* = .047) in the ICBT group. There was 1 suicide attempt in the ISUPPORT group; no other serious adverse events occurred in either group.

**Conclusions and Relevance:**

In this randomized clinical trial, internet-delivered cognitive behavioral therapy was an efficacious and cost-effective intervention for children and adolescents with SAD. Implementation in clinical practice could markedly increase the availability of effective interventions for SAD.

**Trial Registration:**

ClinicalTrials.gov Identifier: NCT03247075

## Key Points

**Question:**

Is internet-delivered cognitive behavioral therapy (ICBT) an efficacious and cost-effective treatment for youths with social anxiety disorder (SAD)?

**Findings:**

In this randomized clinical trial of 103 children and adolescents with a principal diagnosis of SAD and their parents, a 10-week course of ICBT was efficacious and cost-effective compared with an active comparator.

**Meaning:**

Internet-delivered cognitive behavioral therapy has the potential to overcome common treatment barriers and increase the availability of evidence-based psychological treatments for young people with SAD; policy makers could consider ICBT with minimal therapist support a promising, low-intensity treatment for children and adolescents with SAD.

## Teaser

This randomized clinical trial assesses the efficacy and cost-effectiveness of therapist-guided internet-delivered cognitive behavioral therapy for social anxiety disorder in youths compared with internet-delivered supportive therapy.

## Introduction

Social anxiety disorder (SAD) is a prevalent childhood-onset disorder associated with lifelong impairment and high societal costs.^1,2^ Cognitive behavioral therapy (CBT) is effective for children and adolescents with SAD^3^ and is considered the first-line treatment in clinical guidelines.^4^ However, only approximately 10% of individuals with SAD have reportedly ever been in contact with a health care professional about their social fears, and few have received an effective treatment.^5,6^ Barriers to treatment access include low availability of trained therapists, high treatment costs, long distances to clinics, and the nature of the social anxiety symptoms themselves, which makes it difficult for the individual to seek help.^7^

The digital revolution has brought great opportunities to increase access to evidence-based treatments for mental disorders.^8^ There is increasing support for the efficacy of technology-delivered CBT for anxiety disorders in youths, with effect sizes in the moderate to large range compared with waitlist controls.^9,10^ The efficacy of internet-delivered CBT (ICBT) for adults with SAD has been well established,^11^ but only 1 large randomized clinical trial of ICBT for youths specifically targeting SAD has been conducted, comparing a generic ICBT program suitable for all childhood anxiety disorders and a SAD-specific ICBT program with a waitlist control. Both active treatments were significantly more effective than the waitlist control, but no significant difference was observed between the 2 active treatments.^12^ To our knowledge, no study evaluating ICBT for SAD has yet used an active comparator, which is essential to distill the true effect of a behavioral intervention beyond the effects of practitioner attention and frequent monitoring and reporting of symptoms.^13^ Furthermore, no formal health economic evaluations of ICBT for SAD in youths have previously been conducted. Cost-effectiveness analyses are important when evaluating new interventions to guide policy makers on how to best allocate resources.^8^ This trial aimed to evaluate the efficacy and cost-effectiveness of therapist-guided ICBT for youths with SAD compared with an active comparator. We hypothesized that ICBT would be more efficacious and cost-effective than the active comparator.

## Methods

### Study Design

This single-masked randomized clinical trial (RCT) compared therapist-guided ICBT with therapist-guided internet-delivered supportive therapy (ISUPPORT) for children and adolescents (10-17 years of age) with a principal diagnosis of SAD. The trial was conducted at a clinical research unit integrated within the Child and Adolescent Mental Health Services in Stockholm, Sweden. Recruitment took place from September 1, 2017, to October 31, 2018. Participants (N = 103) were randomized to 10 weeks of ICBT (n = 51) or ISUPPORT (n = 52). The primary end point was set to 3 months after the treatment period (May 2019) because of a previously observed tendency for participants in ICBT trials to report continued improvement beyond treatment termination.^14,15^ Participants randomized to ISUPPORT were offered ICBT after the 3-month follow-up. The trial was approved by the Stockholm Regional Ethical Review Board and publicly registered. Following the Swedish law, younger participants (10-14 years of age) provided verbal assent, whereas older participants (15-17 years of age) and their parents provided written consent. The trial protocol can be found in Supplement 1. Moderation and mediation analyses described in the trial protocol, as well as results from the genetic part of the study, will be reported separately. This study followed the Consolidated Standards of Reporting Trials (CONSORT) reporting guideline.

### Participants

Referrals from health care professionals and self-referrals from families living anywhere in Sweden were accepted. The trial was advertised at the Child and Adolescent Mental Health Services clinics in Stockholm, Sweden, in newspapers, and on social media. Inclusion criteria were as follows: a principal diagnosis of SAD according to the *DSM-5*^16^ criteria; age of 10 to 17 years; ability to read and write Swedish; access to the internet; a parent able to coparticipate in the treatment; and if taking psychotropic medication, having been taking a stable dose for 6 weeks or more before enrollment. Exclusion criteria were as follows: diagnosed autism spectrum disorder, psychosis, bipolar disorder, or severe eating disorder; high risk of suicide; ongoing alcohol or substance abuse; and having received CBT for any anxiety disorder within the last 6 months (defined as ≥5 sessions of CBT including in vivo exposure). Table 1 gives the baseline demographic and clinical characteristics of the participants.

**Table 1.** Baseline Sociodemographic and Clinical Characteristics of the Study Participants Assigned to ICBT or ISUPPORT

Characteristic	ICBT (n = 51)	ISUPPORT (n = 52)	Total (N = 103)
Female, No. (%)	41 (80)	38 (73)	79 (77)
Male, No. (%)	10 (20)	14 (27)	24 (23)
Age, mean (SD), y	13.6 (2.0)	14.5 (2.1)	14.1 (2.1)
Duration of SAD, mean (SD), y	6.2 (3.8)	5.5 (3.3)	5.9 (3.6)
Previous treatment^a^			
SAD	12 (24)	17 (33)	29 (28)
Another disorder	27 (53)	29 (56)	56 (54)
No. of comorbid diagnoses			
0	19 (37)	31 (60)	50 (49)
1	21 (41)	13 (25)	34 (33)
2	7 (14)	5 (10)	12 (12)
≥3	4 (8)	3 (6)	7 (7)
Comorbid diagnoses			
Specific phobia	9 (17)	8 (16)	17 (17)
Generalized anxiety disorder	9 (17)	6 (12)	15 (15)
Major depressive disorder	7 (14)	4 (8)	11 (11)
ADHD/ADD	4 (8)	4 (8)	8 (8)
Dyslexia	3 (6)	2 (4)	5 (5)
Selective mutism	4 (8)	1 (2)	5 (5)
Panic disorder and/or agoraphobia	1 (2)	2 (4)	3 (3)
Separation anxiety disorder	2 (4)	0	2 (2)
OCD and related disorders (eg, BDD and tics)	2 (4)	1 (2)	3 (3)
Medication			
None	47 (92)	47 (90)	94 (91)
SSRI	2 (4)	1 (2)	3 (3)
Other^b^	3 (6)	4 (8)	7 (7)
Parental level of education^c^			
<12 y	3 (6)	4 (8)	7 (7)
12 y	4 (8)	3 (6)	7 (7)
Undergraduate studies/university studies	10 (20)	17 (33)	27 (26)
Graduate degree	31 (61)	23 (44)	54 (52)
Postgraduate degree	3 (6)	5 (10)	8 (8)
Parental occupational status^c^			
Employed or self-employed	46 (90)	49 (94)	95 (92)
Other^d^	5 (10)	3 (6)	8 (8)
Parental HADS score, mean (SD)^c^			
Anxiety	7.24 (4.47)	5.37 (3.52)	6.29 (4.10)
Depression	4.06 (3.17)	3.15 (2.94)	3.60 (3.08)

Abbreviations: ADHD/ADD, attention-deficit/hyperactivity disorder; BDD, body dysmorphic disorder; HADS, Hospital Anxiety and Depression Scale; ICBT, internet-delivered cognitive behavioral therapy; ISUPPORT, internet-delivered supportive therapy; OCD, obsessive-compulsive disorder; SAD, social anxiety disorder; SSRI, selective serotonin reuptake inhibitor.

^a^ Psychological treatment.

^b^ Psychostimulants, hypnotics, or antihistamine.

^c^ The parent mainly responsible for study participation.

^d^ Student, unemployed, retired, or on sick leave.

### Procedures

**Figure 1.**
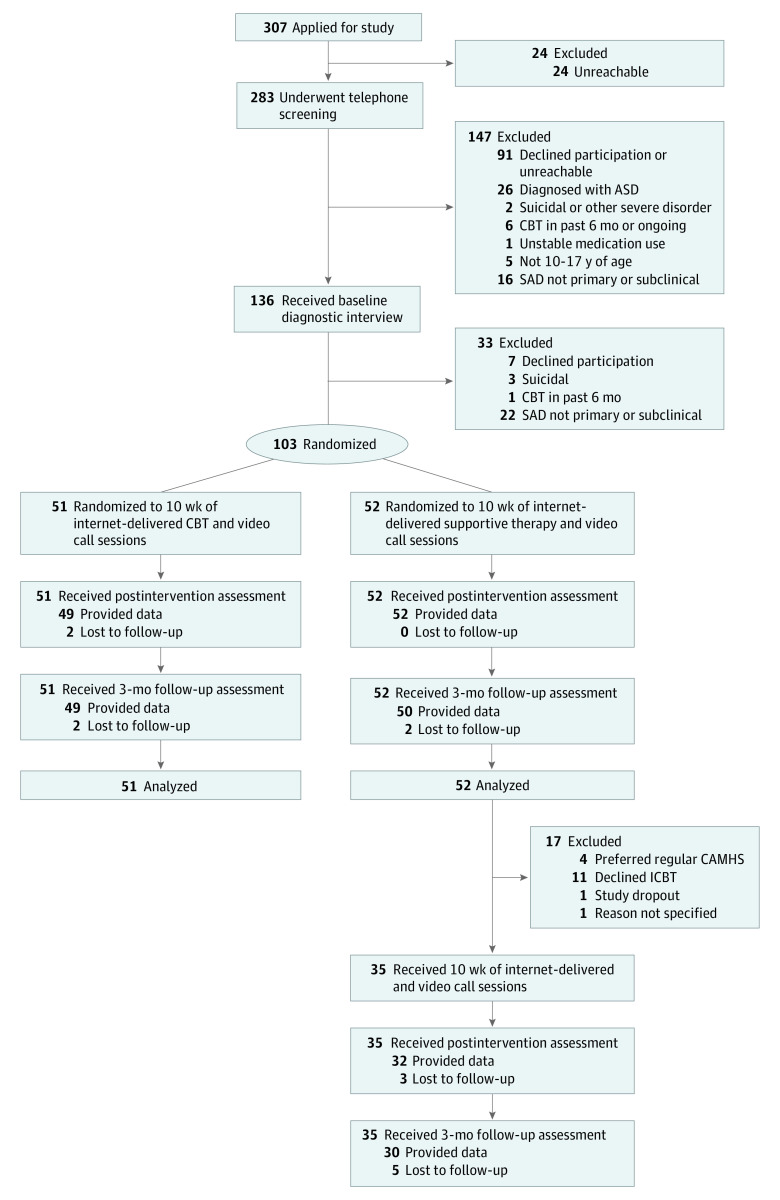
Study Flowchart ASD indicates autism spectrum disorder; CAMHS, Child and Adolescent Mental Health Services; CBT, cognitive behavioral therapy; SAD, social anxiety disorder.

The inclusion procedure consisted of 2 steps: an initial telephone screening and a face-to-face assessment at the clinic. Figure 1 is the CONSORT flowchart. During the face-to-face assessment, a trained psychologist (M.N., T.W., M.J., M.L., and J.H.) administered the Anxiety Disorders Interview Schedule, Child Version (ADIS-C)^17^ jointly with young persons and their parents to ascertain the presence of a diagnosis of SAD or any comorbid disorders according to the *DSM-5*.^16^ Verbal and written information was provided to the youths and the parents.

The ADIS-C diagnostic interview and child- and parent-report measures were administered before treatment, after treatment (10 weeks), and at 3-month follow-up (22 weeks). Trained psychologists masked to treatment allocation conducted the posttreatment and 3-month follow-up assessments.

The random allocation sequence was generated by an independent clinical trials unit, the Karolinska Trial Alliance, in blocks of 4 or 6 and placed in opaque and sealed envelopes. The envelopes were managed by an independent administrator not otherwise involved in the study. An external observer from the Karolinska Trial Alliance also monitored the trial regularly (eMethods in Supplement 2).

### Outcomes

The primary outcome measure was the masked Clinician Severity Rating (CSR) score derived from the ADIS-C interview. The CSR ranges from 0 to 8, with scores of 4 or higher indicating caseness. Secondary outcomes included masked assessor–rated clinical improvement, presence of a SAD diagnosis, global function and treatment adherence, and a range of child- and parent-reported measures of social anxiety, psychopathology, global function, quality of life, treatment credibility, and costs (eMethods in Supplement 2). All child- and parent-report measures were administered via a secure online platform (accessible via personal login, password, and a single-use code) at all time points. The complete lists of measures and assessment points are provided in eResults and eTables 1 and 2 in Supplement 2. Extensive measures were taken to ensure the reliability of measurements, integrity of the masked assessments, and fidelity to the treatment protocols (eMethods in Supplement 2).

### Interventions

The therapist-guided ICBT program is a revised version of the treatment previously developed and piloted by our research group.^18^ The main components are psychoeducation about SAD, gradual exposure to social situations, social skills training, focus shifting (from internal to external attention), reduction of safety behaviors and avoidance, replacement of overly negative thinking with adaptive thoughts, and construction of a plan for relapse prevention.

The active comparator ISUPPORT included psychoeducation about SAD and information about healthy habits (such as the importance of physical activities) and interpersonal relations (such as the significance friendships may have for one’s well-being). Mimicking supportive therapy, ISUPPORT also included encouragement from the therapists to generate and try strategies for handling challenging social situations and to continue to use strategies that the participant found helpful. Crucially, none of the key components thought to constitute the active elements of CBT for SAD (eg, exposure) were included.

In both conditions, three 20- to 30-minute video call sessions were provided at weeks 3, 5, and 7, and parents were provided with parallel access to 5 specifically designed modules, which they accessed through a separate login to the secure online platform (eMethods in Supplement 2).

Dedicated therapists provided asynchronous support throughout the treatment in both groups. For a full description of the secure online platform, the treatment content, and supporting therapists, see the eMethods and eTables 3 and 4 in Supplement 2.

### Statistical Analysis

The power calculation was made in G*power^19^ (assuming repeated measures with within-between interaction). A recent trial^14^ comparing ICBT with an active comparator for mixed anxiety disorders in children found a between-group effect size of *d* = 0.77. However, SAD may be more challenging to treat than other kinds of anxiety disorders^20^; thus, we opted for a more conservative estimate of effect size (*d* = 0.40). A sample size of 101 was calculated to be sufficient using a power of 85%, with a 2-tailed α = .05 and assuming a maximum dropout rate of 10%.

Following the intention-to-treat principle, the primary and secondary outcome analyses included all participants who were randomized. Continuous variables measured at the 3 assessment points (before treatment, after treatment, and 3-month follow-up) were analyzed with linear mixed models with subject-specific intercepts and slopes (random effects), fitted with full maximum likelihood. Linear mixed models use all available data, account for dependence among repeated observations, and provide unbiased estimates in the presence of missing information under a fairly unrestrictive assumption.^21^ Estimates of population mean effect of time and differential change over time as a function of treatment group were determined with a fixed time and time × group interaction effects (with Satterthwaite approximation for denominator degrees of freedom). As a secondary categorical outcome, we estimated the probability that participants in either group would no longer meet diagnostic criteria for SAD at the 3-month follow-up, using a logistic regression analysis, fitted with maximum likelihood with nonnormality robust SEs.

On the basis of model estimates, we computed effect sizes in the form of standardized mean difference between-group effect size (Cohen *d*) for continuous variables and odds ratio (OR) for the categorical variable (ie, remission). The α level was set to *P* < .05 (2-tailed).

Costs were estimated using the Trimbos/iMTA Questionnaire for Costs Associated With Psychiatric Illness–Parent Version^22^ unit cost frequencies multiplied by unit costs (eTables 8 and 9 in Supplement 2). Treatment costs for ICBT and ISUPPORT were estimated from treatment time in hours (for written therapist feedback, video calls, and telephone calls) (eTable 5 in Supplement 2) multiplied by the mean psychologist salary in Sweden (SEK 41 000/month [$4476], reference year 2019). Cost differences at baseline were analyzed using the Wilcoxon rank sum test. Costs were accumulated from pretreatment until the 3-month follow-up. All costs were converted into euros, with the reference year July 2018 to June 2019 and according to the European Central Bank’s conversion rate (10.4523 SEK = €1, $1.1412). Cost-effectiveness analyses were conducted using diagnostic status as the health outcome as well as a cost-utility analysis using Child Health Utility 9D^23^ for estimation of quality-adjusted life-years. Both analyses were conducted from the societal perspective, including all available costs, as well as from the health care professional perspective, including treatment costs (eMethods in Supplement 2). As a global estimate of cost-effectiveness, incremental cost-effectiveness ratios were calculated as follows: (Cost of ICBT – Cost of ISUPPORT)/(Effect of ICBT – Effect of ISUPPORT). Additional details about the statistical analyses, including sensitivity analyses, are described in the eMethods in Supplement 2.

## Results

**Figure 2.**
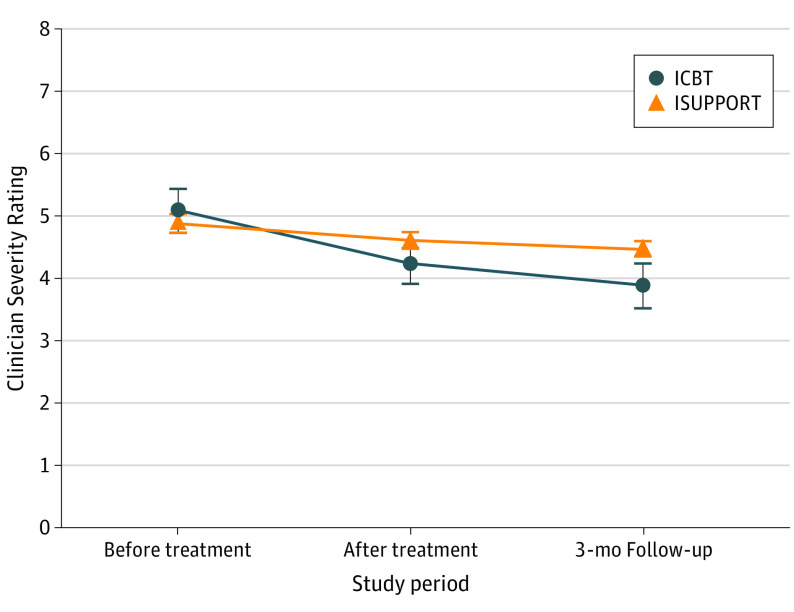
Mean Masked Assessor–Rated Severity of Social Anxiety Disorder Participants randomized to 10 weeks of internet-delivered cognitive behavioral therapy (ICBT) or internet-delivered supportive therapy (ISUPPORT). Error bars indicate SEs.

The sample consisted of 103 youths (mean [SD] age, 14.1 [2.1] years; 79 [77%] female). Table 2 presents the results from the linear mixed models for the primary and secondary outcome measures. The main effect of time was statistically significant for all continuous variables, indicating improvement over time across groups. The within-group effect sizes for the primary outcome measure (CSR scores) from before treatment to the primary end point were large (*d* = 1.24) for ICBT and moderate (*d* = 0.52) for ISUPPORT. Mean (SD) CSR scores for ICBT at baseline and at the 3-month follow-up were 5.06 (0.95) and 3.96 (1.46), respectively, compared with 4.94 (0.94) and 4.48 (1.30) for ISUPPORT. We observed a statistically significant time × group interaction effect (β [SE] = −0.27 [0.09]; *P* = .005), with a model-implied between-group effect size of moderate strength for the primary outcome measure (CSR) (*d* = 0.67; 95% CI, 0.21-1.12) at the primary end point, favoring ICBT (Figure 2). Statistically significant time × group interaction effects were also observed on all continuous secondary outcomes, with the only exception being quality of life (Child Health Utility 9D) (β [SE] = −0.58 [0.51]; *P* = .26). In all cases, results favored ICBT, and most between-group effect sizes at 3-month follow-up were in the moderate range (*d* = 0.64; 95% CI, 0.27-1.01 for child-reported SAD symptoms; *d* = 0.83; 95% CI, 0.43-1.22 for parent-reported SAD symptoms; *d* = 0.47; 95% CI, 0.07-0.88 for child-reported depressive symptoms; *d* = 0.78; 95% CI, 0.38-1.17 for parent-reported anxiety and depressive symptoms; *d* = 0.39; 95% CI, 0.03-0.74 for masked assessor–reported global functioning; and *d* = 0.48; 95% CI, 0.08-0.89 for parent-reported general functioning) (Table 2). For completeness, we report the outcomes from the posttreatment assessment in eTable 6 in Supplement 2 and the results for participants crossing over from ISUPPORT to ICBT after the 3-month follow-up in the eResults and eTable 7 in Supplement 2.

**Table 2.** Results From the Linear Mixed-Effects Models and Effect Sizes at the 3-Month Follow-up^a^

Outcome	ICBT		ISUPPORT		Linear mixed model		Unstandardized mean difference (95% CI)	Effect size at 3-mo follow-up
	No.	Mean (SD)	No.	Mean (SD)	β (SE)	*P* value		*d* (95% CI)
Masked assessor–rated SAD severity (CSR)								
Before treatment	51	5.06 (0.95)	52	4.94 (0.94)	NA	NA	NA	NA
3-mo Follow-up	49	3.96 (1.46)	50	4.48 (1.30)	−0.27 (0.09)	.005	−0.62 (−1.04 to −0.20)	0.67 (0.21 to 1.12)
Child-reported SAD symptoms (LSAS-C)								
Before treatment	51	85.25 (24.54)	52	77.44 (28.45)	NA	NA	NA	NA
3-mo Follow-up	43	63.02 (28.69)	43	75.93 (28.93)	−6.99 (2.06)	.001	−16.40 (−25.88 to −6.91)	0.64 (0.27 to 1.01)
Parent-reported SAD symptoms (LSAS-P)								
Before treatment	51	96.22 (21.87)	52	86.02 (26.68)	NA	NA	NA	NA
3-mo Follow-up	45	70.38 (32.10)	51	82.10 (34.03)	−8.72 (2.13)	<.001	−20.46 (−30.25 to −10.67)	0.83 (0.43 to 1.22)
Child-reported depressive symptoms (RCADS-C-dep)								
Before treatment	51	4.35 (3.07)	52	3.92 (2.71)	NA	NA	NA	NA
3-mo Follow-up	43	2.93 (3.07)	43	3.91 (3.08)	−0.58 (0.25)	.02	−1.36 (−2.53 to −0.19)	0.47 (0.07 to 0.88)
Parent-reported anxiety and depressive symptoms (RCADS-P)								
Before treatment	51	45.96 (17.20)	52	39.54 (14.89)	NA	NA	NA	NA
3-mo Follow-up	45	32.18 (20.01)	51	39.08 (20.38)	−5.59 (1.45)	<.001	−13.11 (−19.78 to −6.44)	0.78 (0.38 to 1.17)
Masked assessor–rated global functioning (CGAS)								
Before treatment	51	54.51 (7.31)	52	56.96 (9.47)	NA	NA	NA	NA
3-mo Follow-up	49	59.45 (10.55)	50	58.88 (9.55)	1.38 (0.64)	.03	3.26 (0.26 to 6.19)	0.39 (0.03 to 0.74)
Parent-reported general functioning (WSAS-P)								
Before treatment	51	14.65 (7.14)	52	13.04 (6.96)	NA	NA	NA	NA
3-mo Follow-up	45	10.47 (8.47)	51	13.14 (9.06)	−1.43 (0.62)	.02	−3.35 (−6.18 to −0.53)	0.48 (0.08 to 0.89)
Child-reported quality of life (CHU9D)								
Before treatment	51	12.69 (6.25)	52	12.71 (6.42)	NA	NA	NA	NA
3-mo Follow-up	43	8.70 (6.89)	43	10.35 (6.02)	−0.58 (0.51)	.26	−1.35 (−3.69 to 0.99)	0.21 (0.15 to 0.57)

Abbreviations: CGAS, Children’s Global Assessment Scale; CHU9D, Child Health Utility 9D; CSR, Clinician Severity Rating; ICBT, internet-delivered cognitive behavioral therapy; ISUPPORT, internet-delivered supportive therapy; LSAS-C, Liebowitz Social Anxiety Scale, Child Version; LSAS-P, Liebowitz Social Anxiety Scale, Parent Version; NA, not applicable; RCADS-C-dep, Revised Children’s Anxiety and Depression Scale, Child Version, depression; RCADS-P, Revised Children’s Anxiety and Depression Scale, Parent Version; SAD, social anxiety disorder; WSAS-P, Work and Social Adjustment Scale, Parent Version.

^a^ Fixed-effects parameter estimates β (SE) represent the time × group interaction for continuous outcomes, with all randomized individuals (n = 103). Positive effect sizes (*d*) indicate results favoring ICBT. Means (SDs) are observed values.

The observed proportion of SAD-free participants was larger in the ICBT group than in the ISUPPORT group at the 3-month follow-up (30.6% vs 18.0%). However, the difference was not statistically significant (OR, 2.44; 95% CI, 0.88-6.75; *P* = .09). Treatment completion, adherence, satisfaction, and credibility are reported in the eResults and eTable 5 in Supplement 2.

The interrater reliability was excellent for the CSR before treatment (intraclass correlation coefficient = 0.91; 95% CI, 0.55-0.97) and very good for the presence of a SAD diagnosis before treatment (κ = 0.86). Treatment fidelity during the video call sessions was excellent because the external assessors noted no protocol violations. Masking integrity checks found that masked assessors were slightly better than chance at guessing the participants’ group allocation, but no significant difference was found in SAD severity at the 3-month follow-up between participants whose group allocation was guessed correctly compared with those whose allocation was guessed incorrectly (*t*_96_ = 0.12, *P* = .91). For more details, see the eResults in Supplement 2.

One suicide attempt was reported and managed in ISUPPORT. No other serious adverse events were reported in either condition (eResults in Supplement 2).

**Figure 3.**
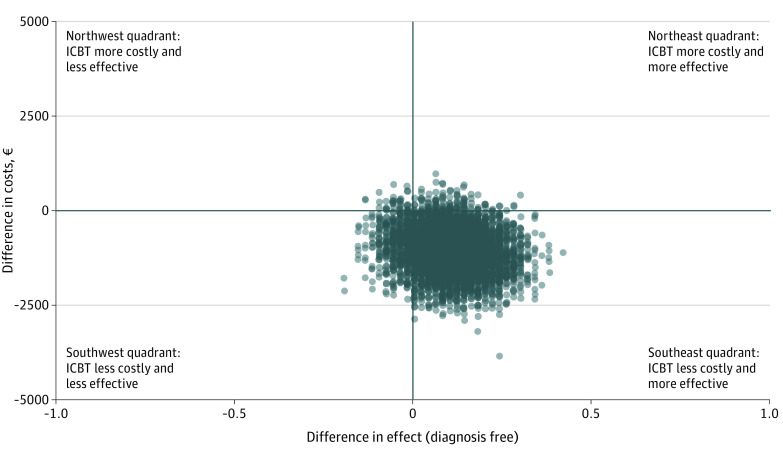
Cost-effectiveness Plane for Societal Cost and Diagnostic Status Differences A concentration of dots in the southeast quadrant indicates a higher probability that internet-delivered cognitive behavioral therapy (ICBT) is more cost-effective than internet-delivered supportive therapy (ISUPPORT); €1 = $1.1412.

Before treatment, no statistically significant between-group difference was found in health-related costs (eg, health care consumption and school absenteeism) (*z* = 0.77, *P* = .44). During the period from before treatment to 3-month follow-up, the average societal cost was €2426.2 (95% CI, €1805.7-€3046.7) ($2768.80 [95% CI, $2060.70-$3476.90]) for the ICBT group and €3502.5 (95% CI, €2543.0-€4462.1) ($3997.10 [95% CI, $2902.10-$5092.10]) for the ISUPPORT group. The incremental cost-effectiveness ratio regarding total societal cost differences and differences in remitter status was −€17 900.7 (95% CI, −€19 493.9 to −€16 307.5) (−$20 428.30 [95% CI, −$22 246.40 to −$18 610.10]), indicating that ICBT was associated with cost savings while generating more participants free of SAD compared with ISUPPORT. The cost-effectiveness plane for societal cost and diagnostic status differences is presented in Figure 3. Subtotal cost analyses demonstrated that the main drivers of the cost differences between the groups were decreases in medication use (*z* = 2.38, *P* = .02) and decreases of school productivity losses (*z* = 1.99, *P* = .047) in the ICBT group (eFigure 4 in Supplement 2). School productivity loss refers to days that the child attends school but performs suboptimally because of social anxiety (eResults in Supplement 2). Additional cost-effectiveness results are presented in eResults and eFigures 1, 2, and 3 in Supplement 2.

## Discussion

In this randomized clinical trial, therapist-guided ICBT was significantly more efficacious than ISUPPORT in reducing social anxiety symptoms, as well as depression, anxiety, and functional impairment, with between-group effect sizes in the moderate range. From a societal perspective, ICBT was cost saving, with the main drivers of the savings being explained by a greater decrease in medication use and an increase in school productivity for youths receiving ICBT compared with those receiving ISUPPORT. From a health care professional perspective, ICBT was more costly because of longer mean therapist support times but also more effective than ISUPPORT. Only 1 serious adverse event was recorded (a suicide attempt in the ISUPPORT group).

The characteristics of the sample are similar to those in previous face-to-face trials of CBT for SAD regarding age and sex composition,^16^ symptom severity,^24,25^ frequency of comorbid disorders,^26,27^ and duration of SAD symptoms.^28^ For the ICBT group, a large within-group effect size was observed on the primary outcome measure, which is comparable to the results of a previous ICBT trial.^12^ Child- and parent-reported symptoms of social anxiety were reduced by more than 25%, corresponding to a large effect. The proportion of SAD-free participants (30.6%) was similar to that of previous ICBT trials of youths^12^ and adults^29^ with SAD but lower than in previous face-to-face CBT trials (approximately 50%-60%).^26,30^ Guided self-help treatments such as ICBT should not be regarded as a substitute for the criterion standard face-to-face treatment but rather a low-intensity treatment to be deployed as a first step, thus freeing resources for young people who require more intensive treatments.

A previous ICBT trial^14^ of children with mixed anxiety disorders, including SAD, found that ICBT was cost-effective, with the main cost saving being reduced health care costs. In the current trial, the main cost savings originated from reduced medication use and increased school productivity (parent-reported measure of the child’s ability to perform on days when he/she is present in school).

Going forward, some modifications could be considered regarding, for example, treatment length and therapist support. Because young persons with SAD seem to require more time to remit than those with other anxiety disorders, ICBT may require longer periods and/or intensified therapist support. Furthermore, parental involvement in ICBT may be particularly important for preadolescents and younger children to engage in the treatment.^31^ The optimal amount of therapist support and parental involvement in ICBT, in relation to child age, should be formally investigated. Identifying clinically useful variables associated with ICBT outcome would be valuable to identify and target more specific mechanisms that may be involved in the maintenance of SAD. In addition, dismantling studies are needed to better understand what components of ICBT contribute the most to the observed reduction of social anxiety. Because SAD often occurs with comorbid anxiety disorders (37% of youths in the current sample), it would also be important to compare disorder-specific forms with transdiagnostic forms of ICBT with regard to their efficacy on comorbid conditions. Furthermore, long-term follow-ups are warranted to establish the durability of the results. Participants in the current trial will be followed up 12 months after treatment, but the results will be presented separately.

### Strengths and Limitations

This study has several strengths. First, the use of an active comparator effectively controlled for nonspecific aspects of treatment. Second, extensive measures were taken to ensure the reliability of the measurements, integrity of the masked assessments, and adherence to treatment protocols. Third, participant retention was high and data loss minimal (<4% missing on the primary outcome measure). Fourth, the trial was externally monitored.

The study also has limitations. One is that most participants were self-referred and could therefore have been more motivated to work with the mostly self-guided treatment compared with typical patients with SAD seen in Child and Adolescent Mental Health Services. Another limitation is that despite best efforts to mimic the format of the active treatment and to make the control treatment as convincing and relevant as possible, the comparator ISUPPORT was seen as somewhat less credible than ICBT. The difference in credibility may have affected treatment expectations, although post hoc analyses (eResults in Supplement 2) found that credibility was not associated with the number of completed modules, total therapist time, change in CSR score, or diagnostic status at the 3-month follow-up. Thus, the slightly superior credibility associated with ICBT did not seem to explain why participants in this group improved more.

## Conclusions

In this randomized clinical trial of children and adolescents with a principal diagnosis of SAD and their parents, a 10-week course of ICBT was efficacious and cost-effective compared with an active comparator. Internet-delivered cognitive behavioral therapy has the potential to overcome common treatment barriers and increase availability of evidence-based psychological treatments for this patient group.

## Notes

Supplement 1. Trial Protocol

Supplement 2. eMethods eResults. eReferences **eTable 1.** List of Measures **eTable 2.** Overview of Assessment Points **eTable 3.** Treatment Content for Ten Weeks of ICBT **eTable 4.** Treatment Content for Ten Weeks of ISUPPORT **eTable 5.** Treatment Completion, Credibility, Satisfaction and Adherence as Well as Therapist Time **eTable 6.** Observed Means, Standard Deviations and Model-Implied Standardized Mean Difference (Effect Size d) of Primary and Secondary Outcomes at Post Treatment **eTable 7.** Secondary Outcomes for Participants Receiving ICBT 3-Months After ISUPPORT. Analysis Includes Complete Cases **eTable 8.** Unit Costs and Sources **eTable 9.** Resource Use Frequencies **eFigure 1.** Cost-effectiveness Plane Regarding Societal Cost and QALYs (Quality Adjusted Life Years) **eFigure 2.** Willingness-to-Pay Graph Depicting the Probability of ICBT to Be Cost-effective Compared to ISUPPORT: Societal Perspective (Total Costs and Diagnostic Status) **eFigure 3.** Willingness-to-Pay Graph Depicting the Probability of ICBT to Be Cost-effective Compared to ISUPPORT. Health Care Provider Perspective (Treatment Costs and Diagnostic Status) **eFigure 4.** Sub-total Cost Differences Between ICBT and ISUPPORT During the Period From Baseline to 3-Month Follow-up. *p < 0.05

Supplement 3. Data Sharing Statement.
