# Supplementary material for: Therapist-Guided Internet-Delivered Cognitive Behavioral Therapy vs Internet-Delivered Supportive Therapy for Children and Adolescents With Social Anxiety Disorder: A Randomized Clinical Trial
Source: JAMA Psychiatry. 2021 May 12;78(7):1–9. doi: 10.1001/jamapsychiatry.2021.0469 (PMC8117054; doi:10.1001/jamapsychiatry.2021.0469)
Supplement: Supplement 1. — Trial Protocol [file jamapsychiatry-e210469-s001.pdf]

THERAPIST-GUIDED INTERNET-DELIVERED COGNITIVE BEHAVIOURAL THERAPY VERSUS INTERNET-DELIVERED SUPPORTIVE THERAPY FOR CHILDREN AND ADOLESCENTS WITH SOCIAL ANXIETY DISORDER: A RANDOMISED CONTROLLED TRIAL

**Long title of the trial**	Therapist-guided internet-delivered cognitive behavioural therapy versus internet-delivered supportive therapy for children and adolescents with social anxiety disorder: a randomised controlled trial
**Short title of trial**	ICBT for youth with social anxiety disorder
**Version and date of protocol**	1.3, 2017-09-18
**Sponsor**	Child and Adolescent Mental Health Services, Stockholm Region
**Funder (s)**	The Swedish Research Council for Health, Working Life and Welfare (Forte 2014-4052) and Region Stockholm (HNSV 14099). JH was supported by Region Stockholm (Dnr: K0173-2016; clinical postdoctoral appointment: 2017-2020). ES was supported by Region Stockholm (clinical research appointment; 2017-0605).
**Trial registration number**	NCT03247075
**Site**	Stockholm, Sweden
**Principal investigator**	Eva Serlachius, Associate professor, Department of Clinical Neuroscience, Karolinska Institutet
**Sponsor representative**	Göran Rydén, Director of Operations, MD, the Child and Adolescent Mental Health Services, Region Stockholm

Version history

Version number	Version date	Reason for change
1.1	17/08/2017	First version
1.2	08/09/2017	1. SPWSS removed due to decision to use the more specific process measures.<br>2. KIDSCREEN-10 removed (Swedish translation of CHU9D was completed and used instead).<br>3. FASA-C removed to reduce load on children and adolescents.<br>4. Treatment length adjusted from 12 to 10 weeks for both conditions.
1.3	29/09/2017	5. RCADS-C was removed to reduce the load on children and adolescents. Only RCADS depression subscale was kept for youths.<br>6. WSAS-C was removed to reduce the load on children and adolescents.

Protocol version number: 1.3

Eva Serlachius Principal Investigator (PI)

Date 16/06/20

Signature

Eva Serlachius

Study protocol

INTERNET-DELIVERED COGNITIVE BEHAVIOR THERAPY VERSUS INTERNET-DELIVERED SUPPORT AND COUNSELING FOR YOUTH WITH SOCIAL ANXIETY DISORDER – A RANDOMIZED CONTROLLED TRIAL

Table of contents

Presentation of research group 5
Study site 5
Background 7
Background for additional study domains 8
In conclusion 9
Objective 9
Method 10
Subjects 10
Measures 11
Clinician administered measures 11
Parent and self-report measures of anxiety 12
Other parent and self-report measures 13
Demographic and background variables 14
Procedure 15
Telephone interview 16
Face-to-face baseline diagnostic assessment 17
Baseline self-and parent report measures 17
Self-rating assessments during intervention 17
Safety procedures 17
Outcome measure reliability procedures 17
Post-treatment and follow-up measurement 18
Non-responders 20
Genotyping 20
Register linkage 20
Intervention 21
Statistical analysis 22
Project schedule 23
Anticipated effects 23
Ethical considerations 23
Presentation of results 23

PRESENTATION OF RESEARCH GROUP

The members of the research group have extensive experience from clinical work with children and adolescents with anxiety disorders as well as research and clinical implementation of internet-delivered CBT treatment. All investigators have at least 50% planned research time.

Eva Serlachius, associate professor at Karolinska Institutet, is a child psychiatrist and principal investigator. She is also responsible for the integration of the project into the child and adolescent psychiatry clinic in Stockholm.

Martina Nordh, PhD candidate, licensed psychologist, has extensive experience from working with CBT for child psychiatric disorders, as well as from ICBT-treatments for anxiety disorders and will be responsible for the development of the ICBT treatment protocol and the protocol for the control condition. Martina will also be the study coordinator for the current trial.

Jens Högström, PhD, licensed psychologist, has extensive experience from clinical trials on ICBT for youths with anxiety disorders, OCD and conduct problems.

David Mataix-Cols, Professor at Karolinska Institutet, accomplished OCD and related disorders researcher with extensive trial experience in children and adolescents.

Sarah Vigerland, PhD, licensed psychologist, has long experience from trials investigating ICBT for youths and will provide expertise on the technical platform used to administer ICBT.

Maral Jolstedt, PhD candidate**,** licensed psychologist, has substantial experience from treating children and adolescents with anxiety disorders with CBT and develop ICBT for children with anxiety disorders.

Tove Wahlund PhD candidate**,** licensed psychologist, has long experience from treating adults with psychiatric disorders with CBT, as well as developing ICBT for, and treating, adolescents with excessive worry and children with anxiety disorders.

Thalia Eley, Professor at King´s College London, is a senior researcher in the field of genetics and therapygenetics, and will be responsible for the genetics part of the study.

STUDY SITE

The project is a collaboration between the Department of Clinical Neuroscience at Karolinska Institutet, the Child and Adolescent Mental Health Service in Stockholm (Barn- och ungdomspsykiatrin i Stockholm) and King´s College London.

All study participants are assessed and treated at the Child and Adolescent Mental Health Service in Stockholm, a part of the Healthcare Provision Stockholm County (SLSO) and the clinic is located within the premises of CAP Research Center, Gävlegatan 22, 113 30 Stockholm, Sweden.

BACKGROUND

Social anxiety disorder (SAD): SAD is characterized by an intense fear of being scrutinized and negatively evaluated in social or performance situations. The individual is typically afraid of exhibiting unacceptable behaviors and to show signs of anxiety, such as blushing or trembling and may therefore avoid social situations or endure them under intense distress[1]. The disorder is one of the most common mental disorders among youth with a 12-month prevalence of 3.4% [2] and 8.6% experiencing the disorder at some point in their lifetime[3]. If the disorder is left untreated it may become chronic [3] and can lead to severe secondary effects on wellbeing and functioning, such as increased risk of depression [4] and substance- and alcohol dependence [5] and an increased degree of suicidality and suicide attempts [6]. Considering the substantial distress for youth suffering from SAD, and the burden on their families, paired with long-term societal costs [7, 8], early identification and treatment is imperative.

Cognitive behavioral therapy (CBT): CBT for SAD is effective for adults [9] as well as children and adolescents [10, 11] and is the first-line treatment according to the Swedish National Board of Health [12], as well as international clinical guidelines (the National Institute for Health and Care Excellence; NICE) [13]. Common treatment components in CBT for children and adolescents include psychoeducation, graded exposure in vivo, cognitive restructuring, coping strategies such as relaxation, social skills training and problem solving [14]. Most often parents are included in the treatment to some extent, even though the level of participation can vary from basic information about the condition and treatment program, to active participation as a “co-therapist” with training in behavior management [14]. But despite effective treatment and the high level of impairment caused by the disorder, only a small proportion of youth with social anxiety seek help for their problems [15, 16] and even fewer receive effective treatment [17]. Barriers to receiving evidence-based and effective treatment include limited availability of trained therapists but also stigma related to visiting a psychiatric clinic and practical issues such as long travelling distances between home and clinic, and having to take time off school or work during daytime opening hours of the clinic.

Internet-delivered CBT (ICBT): ICBT has been suggested a possible solution to some of these barriers as it

may offer the same treatment components as standard CBT but with the patients working with the online material and home work assignments whenever and wherever suitable. A therapist commonly guides the patient through e-mail. This makes the treatment more accessible as the therapist and patient does not have to work with the treatment at the same time. Furthermore, it may increase treatment capacity, as therapist time per patient often is low. For adults with SAD, ICBT has been established as an evidence-based treatment [18] and as being equally effective as face-to-face CBT [19]. For youth, ICBT has been shown to be effective for mixed anxiety disorders when compared to a waitlist control [20-22] and with similar effects as face-to-face CBT [23]. A recent Australian study [24], compared a generic ICBT-programme (CBT-GEN) and a SAD-specific ICBT-progamme (CBT-SAD) to a waitlist control (WLC) for youth with SAD, where both active treatments were significantly more effective than the waitlist control. However, at post-treatment, only 12.8% and 14.6% for CBT-SAD and CBT-GEN respectively (3.3% for the waitlist control) were free of their SAD-diagnosis. Even though higher remission rates were demonstrated at the 6-month follow-up (CBT-SAD; 29.8% and CBT-GEN 35.4% respectively), this might indicate that solely offering ICBT might not be sufficient for this population. Our research group recently evaluated an ICBT treatment for adolescents with SAD that was supplemented with three group-exposure sessions at the clinic. This *blended* treatment (internet and face-to-face) produced remission rates of 47% at post-treatment and 57% at 6-month follow-up, comparatively more effective than the Australian study [25]. However, adding face-to-face sessions at the clinic restricts the accessibility of the treatment, and compromises the anticipated cost-effectiveness of internet-based treatment. Therefore the current trial will evaluate ICBT supplemented with telephone / Skype support, aiming to provide continuous help with challenging parts of the treatment and to closely monitor participants progress through the treatment.

BACKGROUND FOR ADDITIONAL STUDY DOMAINS

Therapygenetics, gene expression and etiology of social anxiety: In recent years there has been an increased interest in identifying biomarkers that predict treatment response. A number of studies have investigated genetic predictors of therapy response in anxiety disorders and depression, a field that has been termed “therapygenetics” [26]. It has for instance been suggested that individuals who are genetically predisposed to developing psychiatric disorders in negative environments (e.g. stressful life events) may also be more likely to benefit from positive environments [27], and hence may benefit more from therapeutic interventions. However, previous studies have been very limited by their reliance on candidate genes as the candidate approach is restricted by its reliance on prior information about likely pathways, which is relatively minimal. In contrast, a genome-wide approach allows for the exploration of genes across the entire genome but the required sample sizes for these analyses very large. Therefore, to contribute to the general understanding of genetic influence on the etiology of anxiety disorders, as well as the genetic impact on treatment outcome, a collaboration

with Professor Thalia Eley at King´s College London has been established and all participating youth in the study will be asked to provide a saliva sample from which DNA will be extracted. Part of the extracted DNA will be transported to, stored and analyzed at, the Social, Genetic & Developmental Psychiatry Centre (SGDP) – Institute of Psychiatry, King’s College London. Thalia Eley has been collaborating with 10 other research groups (from e.g. the United States, Norway, Germany and Australia) who also conducted CBT-related research and who sent extracted DNA from study participants who had received CBT for anxiety disorders. This collaboration and sharing of biological material is set up in order to eventually enable Genome Wide Association Studies (to establish associations between gene variations and the development of an anxiety disorder), as well as therapygenetics studies with a genome-wide approach (where the association between gene variations and the effect of CBT is evaluated).

IN CONCLUSION

Social anxiety disorder among youth is highly prevalent and causes significant impairment in the lives of the affected. In spite of CBT being the most effective treatment, evidence suggests that many young people with this disorder do not have access to good-quality CBT. Internet-delivered CBT is, as numerous prior studies have shown, an effective method to treat psychiatric conditions in adults, but still there is limited knowledge of ICBT for youth. There are but a few controlled studies on ICBT for children and adolescents with anxiety disorders with promising results, conducted in Sweden and Australia. As our recent pilot trial showed that ICBT for adolescents with SAD is acceptable, feasible and potentially effective, this randomized controlled trial will further evaluate the efficacy of ICBT for youth with SAD. Furthermore, the study can contribute with important knowledge about mediating factors involved in treatment of SAD in youth, as well as the long-term effects of ICBT. The study will also include genotyping of participants to further our understanding of the etiology of SAD.

OBJECTIVE

The primary objective of this study is to test the efficacy of internet-delivered CBT for youth (10 – 17 years) with social anxiety disorder. We aim to conduct a randomized controlled trial with $N = 101$ participants (assuming Cohen’s $d = .4$ (between-group effect), power = .85, $\alpha = .05$) which would allow for a 10% drop-out rate, with maintained sufficient power. For the secondary objective 5 (genetic part of the study), data from this study will be merged with data from similar studies conducted within the research group, in order to achieve sufficient power.

First, participants randomized to the active treatment arm will receive guided ICBT for 10 weeks. Participants randomized to a control condition will first receive 10 weeks of internet-delivered

support and counseling and will be offered ICBT for 10 weeks, after the 3-month follow-up. Our primary research question is:

1. **Is guided ICBT effective compared to internet-delivered support and counseling to reduce social anxiety symptoms?**

Secondly, we want to analyze possible mediating variables involved in the change mechanisms of ICBT. Variables such as self-focus, pre- and post event processing, avoidance and safety behaviors will be tested as potential mediators, using the best study-design possible to derive causality in subsequent statistical analyses:

2. **What are the mechanisms of change in ICBT for youth with SAD?**

Third, a follow-up assessment will be conducted 3- and 12 months after treatment termination. Our hypothesis is that most participants will retain treatment gains to follow-up and some will improve further.

3. **What is the long-term efficacy of guided ICBT?**

Fourth, we aim to study the health-economic benefits of guided ICBT, at post measurement and at follow-up, in terms of cost savings for the youth's family and for the health care system:

4. **Is guided ICBT associated with health-economic benefits in families where a child/adolescent has SAD?**

Fifth, participants will be asked to provide saliva samples in order for us to eventually (when a sufficient number of children and adolescents have been included in this and other upcoming trials) study the association between genetic variations and the development of SAD, as well as effect of genes on the outcome of ICBT.

5. **How do individual genetic variations contribute to the development of SAD (etiology) as well as to the outcome of guided ICBT (therapygenetics)?**

METHOD

Subjects

Inclusion criteria:

- A principal diagnosis of social anxiety disorder, as defined by DSM-5 [28]
- Age between 10 and 17 years
- Ability to read and write Swedish
- Daily access to the internet through a computer or similar device

- A parent or caregiver that is able to co-participate in the treatment
- Participants on psychotropic medication must have been on a stable dose for the last 6 weeks prior to baseline assessment

Exclusion criteria:

- Diagnosed with autism spectrum disorder, psychosis, bipolar disorder or severe eating disorder
- Present risk of suicide
- Ongoing alcohol or substance abuse
- Occurrence of domestic violence
- Completed CBT for any anxiety disorder within the last 6 months (defined as at least 5 sessions of CBT including in vivo exposure sessions)

Measures

Clinician administered measures

Anxiety Disorder Interview Schedule for DSM-IV, child and parent versions (ADIS C/P) [29] is a semi-structured interview that can be conducted with youth and parents separately to assess presence/non-presence of diagnostic criteria according to DSM-IV, in the youth. The ADIS C/P has shown good to excellent kappa coefficients and excellent test-retest reliability [30], as well as adequate concurrent validity when compared with other measures of anxiety [31]. In this trial the ADIS-C will be used to interview youth and parents jointly.

C*linician Severity Rating* (CSR). The ADIS C/P includes a severity rating for each received diagnosis, ranging from 0 - 8. A score of 3 or lower is considered subclinical symptoms whereas a score of 4 or higher indicates that the criterion for a diagnosis, with regard to severity, is fulfilled. CSR is the primary outcome of this study.

Clinical Global Impression - Improvement (CGI-I) [32] is a brief clinician rating of the patients symptom severity change relative to the baseline assessment. The seven-graded scale ranges from 1 = “very much improved” to 7 = “very much worse”.

Children’s Global Assessment Scale (CGAS) [33] is used by clinicians to assess global functioning (scale 0-100) in children and adolescents, with higher scores indicating higher functioning. It has shown moderate to excellent inter-rater reliability, good stability over time and good concurrent as well as discriminant validity [34, 35].

Internet intervention Patient Adherence Scale (iiPAS) is a clinician-rated measure developed by our research group and an adaptation of the *Patient EX/RP Adherence Scale* (PEAS) [36]. The clinician rates the patients´ adherence to the treatment in five dimensions (work pace, engagement, communication with therapist, motivation to change and login frequency) on a five-point scale (0-4; 0 = "does not work with the treatment"/"is not engaged in the treatment"/"does not communicate with the therapist"/"does not work with presented strategies"/"does not login". 4 = "works with the expected work pace"/"is engaged in the treatment"/"does actively communicate with the therapist"/"works with presented strategies"/"logs in often").

Parent and self-report measures of anxiety

Liebowitz social anxiety scale for children and adolescents (LSAS-CA)[37, 38] is a child/adolescent self-report measure of social anxiety. The 24 items measure fear and avoidance in social (12 items) and performance (12 items) situations. For each item the child/adolescent rates the intensity of fear, on a 4-point Likert scale ranging from 0 = "None" to 3 = "Severe", as well as the frequency of avoidance, on a 4-point Likert scale ranging from 0 = "Never" to 3 = "Usually". The scale has shown good psychometric properties, with high internal consistency [39, 40] for the total score as well as the subscales and good construct validity[38]. The parent version has been adapted from LSAS-CA by our research group to be used in this trial.

Revised Children´s Anxiety and Depression Scale – Child and Parent version (RCADS-C/P) [41] (a revised version of the Spence Child Anxiety Scale – SCAS) is a child and parent self-report measure of anxiety- and depression related psychopathology. The 48 items of the RCADS measures five domains of anxiety: generalized anxiety, panic anxiety, separation anxiety, social anxiety and obsessive-compulsive anxiety, as well as symptoms of depression. The four-graded scale ranges from 0 = "Never" to 3 = "Always". The RCADS C/P has been shown to hold strong psychometric properties, with high internal consistency for the total score as well as the subscales, satisfactory test-retest reliability and good criterion validity [42, 43].

Anticipatory social behaviors scale (ASBQ) [44] is a 12-item self-measure questionnaire adapted from the Social Behaviour Questionnaire [45]. Each item measures anxious thoughts *before* a social situation and is scored on a four-point scale ("Never" to "Always"). The questionnaire has a high internal consistency (Cronbach's alpha=0.88) [44]

Post-event processing questionnaire revised (PEPQ-R) [46] is a self-rated measure of anxiety and rumination *after* a social situation. The 8-item version is revised from the Post-event processing questionnaire (PEP-Q)[47]. A visual analogue scale (VAS) is used to rate how much (ranging from 0 = *not at all* to 100 = *totally* agree) the participant engaged in any of the 8 PEP activities (e.g. "After the event was over, did you find

yourself thinking about it a lot?”). The PEPQ-R has excellent internal consistency (Cronbach’s alpha = 0.87)[24, 46].

Focus of attention questionnaire (FAQ) [48] is a self-rated measure of self-focus and external focus in social sitatuations. Each of the 10 items are rated on a 5-point scale ranging from *Not at all* to *Totally* according to how much the participant’s attention matched the item description. Previous studies have reported acceptable to high internal consistency in youth samples [49, 50]

The Subtle Avoidance Frequency Examination (SAFE) [51] is a self-rated measure of safety behaviors in social situations. Each of the 32 items are rated on a 5-point scale ranging from *Never* to *Always* according to how much the participant engages in the different safety behaviors. The SAFE has good construct validity [51] and good internal consistency (Cronbach’s alpha = 0.89) [52].

Other parent and self-report measures

The Child Health Utility 9D (CHU 9D) [53] is a self-rated measure of health-related quality of life. The scale contains nine dimensions with five levels representing increasing degrees of severity within each dimension.

Client Satisfaction Questionnaire – child and parent version (CSQ-C/P) [54]. The CSQ is a 8-item self- and parent rated scale measuring different aspects of satisfaction with treatment.

Treatment credibility and expectancy – child and parent version (C-scale) [55]. A 4-item scale measuring the degree to which participants rate their expectancies regarding various aspects of the intervention.

Education, Work and Social Adjustment Scale – parent version (EWSAS-C/P) [56] is a 5-item parent-rating scale of impaired functioning in psychiatric patients.

Hospital Anxiety and Depression Scale (HADS) [57]. The HADS is a 14-item measure for adults with two subscales, assessing depression and anxiety, respectively (i.e., parents´ level of anxiety and depression in the context of this study). Each item is rated on a 4-point scale. The inventory has good to very good construct validity and internal consistency [58, 59].

Alcohol Disorder Identification Test (AUDIT) [60] is a self-rated screening instrument for hazardous and harmful alcohol consumption. Each of the 10 items is scored from 0 to 4, with higher scores indicating higher consumption, more problematic drinking behaviors and more alcohol-related problems.

Drug Use Disorders Identification Test (DUDIT) [61] is a self-rated screening measure for drug-related problems. Each of the 11 items is scored from 0 to 4, with higher scores indicating more severe drug-consumption and drug-related problems.

Family Accommodation Scale-Anxiety (FASA) [62] is a parent-report scale that measures parental accomodation in families of anxious youth. FASA is a nine-item scale that use a 5-point Likert-type scale (from 0 = *Never* to 4 = *Daily*). It has excellent internal consistency (Cronbachs alfa = .85)[63] as well as convergent and divergent validity [62].

Trimbos/iMTA questionnaire for Costs associated with Psychiatric Illness – Child version (TiC-P) [64]. TiC-P is used to measure consumption of health care, costs associated with mental illness and production loss among parents due to psychiatric problems in the child.

The Negative Effects Questionnaire (NEQ) [65] is a 32-item self-report questionnaire for monitoring and reporting treatment related adverse and unwanted events. The questionnaire has been developed to evaluate negative effects in ICBT for adults with SAD and use a 5-point Likert-scale ranging from *"Not at all"* to "*Extremely*".

Demographic and background variables

In the self-report procedure, parents will also be asked to respond to questions pertaining to parental educational level, occupational status, ethnicity of the child, prior treatment history (e.g. previous CBT or medication), response to previous treatments and onset of anxiety symptoms. Information on how the participants got in contact with the study will be registered (e.g., via media, referral or recommendation).

Table 1 gives an overview of the measures that will be used in the study.

Table 1: List of measures

Category	***Clinician assessed***	***Child self-report***	***Parent report***
**Diagnostic status/ comorbidity**	ADIS-C/P		HADS
**Anxiety symptom severity**	CSR<br>CGI-I	LSAS-CA<br>RCADS-C-dep*	LSAS-P<br>RCADS-P
**Alcohol- and substance abuse**		AUDIT<br>DUDIT	
**Process measures**		PEPQ-R<br>ASBQ<br>FAQ<br>SAFE	FASA-P

**Impairment**	CGAS		EWSAS-P
**Quality of life**		CHU-9D	
**Cost-effectiveness**			TiC-P
**Adherence / compliance**	iiPAS		
**Treatment satisfaction/ Treatment credibility**		CSQ-C<br>C-scale-C	CSQ-P<br>C-scale-P
**Adverse events**		NEQ-C	NEQ-P

*= Only RCADS Depression subscale and suicidal ideation item

Procedure

For recruitment, this RCT will be advertised at child and adolescent mental health services and primary care clinics, as well as through newspapers, other media and social media such as facebook. The inclusion procedure will be carried out in two steps: 1) telephone interview and 2) face-to-face assessment at the clinic. Figure 2 gives an overview of the inclusion, assessment and treatment procedure.

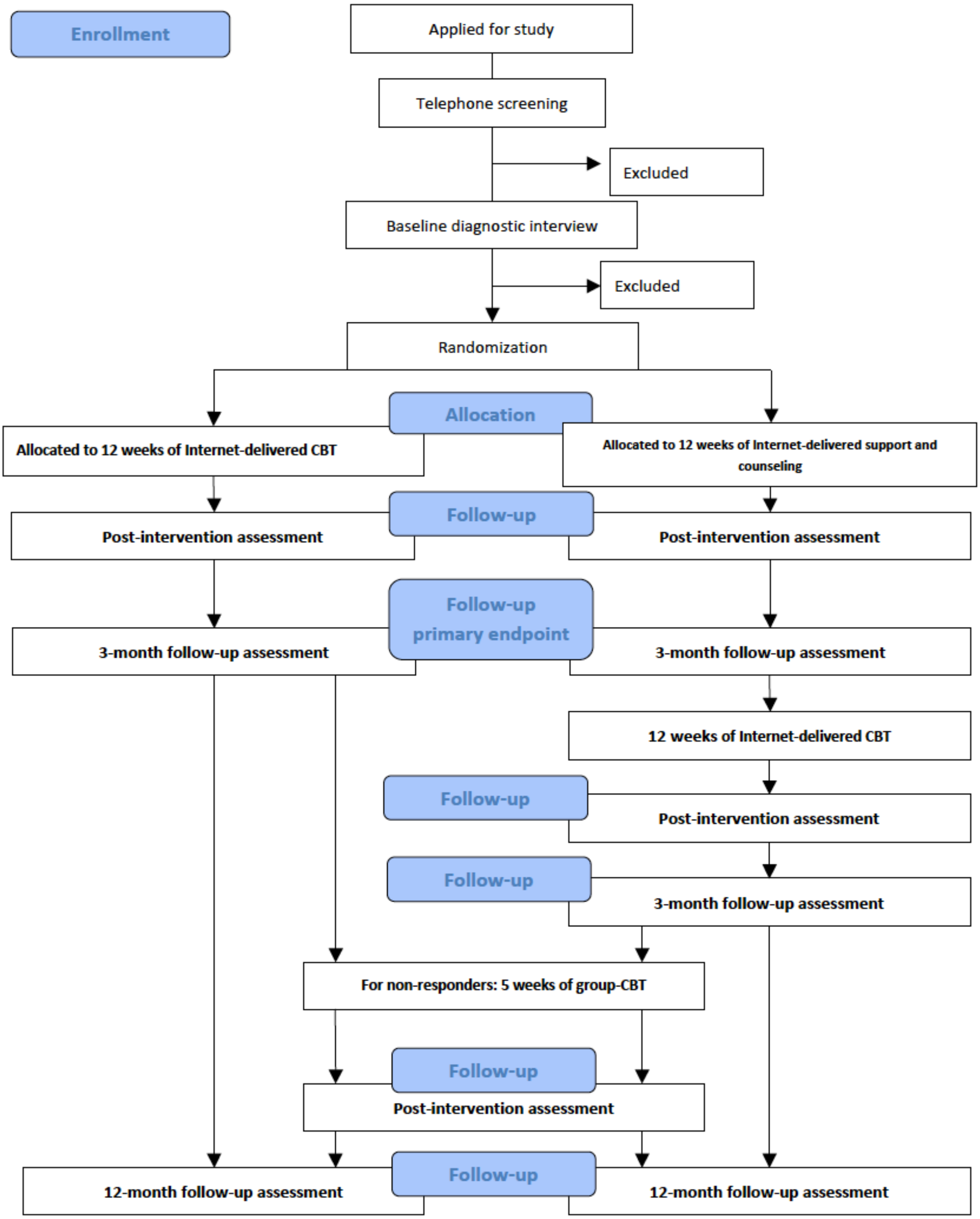

Figure 2: CONSORT study flow chart

Telephone interview

An initial telephone interview will be conducted with the youth/parent in order to broadly assess inclusion and exclusion criteria.

Face-to-face baseline diagnostic assessment

After the telephone screening, the child/adolescent along with his/her parents, are invited to a face-to-face assessment at the Child and Adolescent Mental Health clinic, including the diagnostic screening interview ADIS-C conducted with children/adolescents and parents jointly. If ADIS-C indicates presence of a social anxiety disorder diagnosis, children/adolescents and parents will be invited to participate in the study, provided that the other inclusion criteria are fulfilled and no exclusion criteria prohibits participation. During the face-to-face assessment, participants will be provided with an information sheet and consent form. Adjacent to the baseline assessment, a saliva sample will be collected from those participants who have consented to the genotyping part of the study.

Baseline self-and parent report measures

Participants and parents included in the study after the face-to-face diagnostic assessment are asked to fill in self-report measures on the Internet, provided via personal login and password.

Self-rating assessments during intervention

LSAS-CA, PEPQ-R, ASBQ, FAQ and SAFE are administered to youth at three time points during treatment (after 17, 35 and 53 days respetively) and LSAS-P and are administered to parents at the same time.

Safety procedures

Study participants are in regular contact (on a weekly basis) with experienced clinicians during the ICBT treatment as well as the control condition. Clinicians that suspect any kind of adverse event during the assessment or treatment process will contact the parent via telephone as a first step of assessing the severity of the incident. Adverse events in this context are defined as an actual or potential situation that threatens the patient's well-being, e.g., suicide risk or an acute increase of symptoms. In the case of an adverse event, the clinician informs the study coordinator (MN) for a discussion of adequate action taking. If a continuation of the ICBT treatment or the internet-delivered support and counseling is considered inappropriate with regard to the participants' best interest and well-being, the participant will be excluded from the study and parents/youth will be provided with proper referral information. Any adverse event will be reported in the planned publication of the trial.

Outcome measure reliability procedures

To ensure the reliability of the assessments conducted by study clinicians, all assessors will be trained by experienced instructors in the diagnostic screening instrument ADIS-C. The baseline diagnostic interviews will be recorded (audio/video) and then independently rated in order to estimate inter-

rater coefficients to be reported in the study article. All therapists that participate in the study will also be continuously trained in ADIS-C interviewing, assessment and diagnosing.

Post-treatment and follow-up measurement

Post-treatment measurement with independent clinician rated symptom severity (telephone or face-to-face interview with ADIS-C, CSR, CGI-I and CGAS) as well as self- and parent report measures will be conducted immediately after the intervention (10 weeks after baseline) for both conditions. A follow-up measurement will be performed at 3 months after intervention termination for both conditions. An independent rater blind to treatment condition will conduct the post-treatment assessment, as well as the 3-month follow-up. For participants crossing over from internet-delivered support and counseling to ICBT, the same procedure is repeated after additional 10 weeks (post-treatment) and 22 weeks (3-month follow-up after ICBT). At follow-up, ADIS-C, CSR, CGI-I and CGAS are conducted either over the telephone or face-to-face. For all participants a 12-month follow-up will be conducted one year after finishing the ICBT-intervention, following the same procedure as previous follow-up assessments (however, not a blind assessor). If a joint interview is not possible at post-treatment assessment or at follow-up assessment the parent version of ADIS (ADIS-P) will be used to interview the parent.

Saliva samples will be collected at baseline (pre intervention), from those participants who have consented to the genotyping part of the study.

All self- and parent-report measures will be administered via the Internet at all time points, pre, post and follow-up.

Timeline	Telephone interview	Face-to-face baseline assessment	Pre-treatment self-report measures	After 17 days of ICBT or control	After 35 days of ICBT or control	After 53 days of ICBT or control	Post-treatment assessment 1	Post-treatment assessment 2	3-month follow-up	12-month follow-up
Clinician-rated instruments										
Inclusion / exclusion	X	X								
Demographic data		X								
ADIS-C/P		X					X	X	X	X
CSR		X					X	X	X	X
CGI-I							X	X	X	X
CGAS		X					X	X	X	X
iiPAS					X		X	X	X	X
Self & parent-rated										
Demographic data			X							
LSAS-CA			X	X	X	X	X	X	X	X
RCADS			X*	X*	X*	X*	X*	X*	X*	X*
HADS			X							
EWSAS			X				X	X	X	X
FASA			X	X	X	X	X	X	X	X
FAQ			X	X	X	X	X	X	X	X
ASBQ			X	X	X	X	X	X	X	X
PEPQ-R			X	X	X	X	X	X	X	X
SAFE			X	X	X	X	X	X	X	X
TIC-P			X				X	X	X	X
CHU-9D			X				X	X	X	X
C-scale				X						
CSQ							X	X		
AUDIT			X							
DUDIT			X							
NEQ							X	X		

Table 2. Overview of measures and time points

*Only RCADS Depression subscale and suicidal ideation item will be administered to children

Non-responders

After the 3-month follow-up after ICBT, all non-responders (defined as those participants who either 1) still fulfill diagnostic criteria for their SAD diagnosis and suffer from their SAD-symptoms (CSR>3) 2) have not improved to a clinically meaningful degree (CGI-I>2)) are offered a CBT group-exposure intervention face-to-face at the clinic. The group-exposure sessions builds on a manual previously used in our pilot trial and will consist of 5 group-exposure sessions. Groups are formed of 3-6 participants and the groups will consist of the same members throughout the intervention. Every session is 2 hours long and is led by two therapists. In the event of too few participants accepting the offered treatment or temporal problems to form groups the intervention is offered as individual face-to-face CBT at the clinic. A post-intervention assessment follows the same procedure as previous follow-ups.

Genotyping

Study participants are asked to give saliva samples for DNA extraction. This part is optional and participants can choose not to give samples without any consequences for their participation in the trial and the treatment given. Participants and their parents are informed verbally and in writing about the genotyping part of the study and the procedure of giving saliva samples. The samples will be stored at KI's biobank. In a long-term perspective this material will be part of a bigger effort at our clinic to build a sufficiently large material in order to answer research questions on genetic factors in the etiology of pediatric anxiety disorders. A collaboration with Professor Thalia Eley at King´s College London has been established and a part of the extracted DNA will be transported to, stored and analyzed at, the Social, Genetic & Developmental Psychiatry Centre (SGDP) – Institute of Psychiatry. Thalia Eley has been collaborating with 10 other research groups (from e.g. the United States, Norway, Germany and Australia) who are also conducting CBT-related research and who are sending extracted DNA from study participants who have received CBT for anxiety disorders. This collaboration and sharing of biological material is set up in order to eventually enable Genome Wide Association Studies (GWAS), as well as therapygenetics studies, where large samples are required.

Register linkage

Register data from several Swedish registers (se Table 3) are linked together using the unique personal identification number. The aim with register linkage is to study risk factors for disease (in combination with genetic risk factors), consequences of anxiety disorders and long term outcome of treatment.

Register name	Register holder	Key data
National Patient Register	EpC	Diagnosis, Hospitalization, Suicide attempts
Causes of Death	EpC	Causes of Death
Prescription register (Läkemedelsregistret)	EpC	All presciptions
Multigeneration register	SCB	Linkage between generations
Marital status changes, county changes and immigration/emigration	SCB	
LISA (Longitudinal integration database for health insurance and labour market studies)	SCB	Income, Highest level of education and employment status
Census, FOB (Folk- och bostadsräkningen)	SCB	http://www.scb.se/Pages/List 257507 .aspx
Register of school grades (elementary school and gymnasium)	SCB	Grades
Sick leave, disability	Försäkringskassan	Sick leave, disability pension
VAL data bases: primary care	Karolinska Inst	Diagnoses
The Medical Birth Register	EpC	Birth weight, APGAR
VAL data bases: psychiatric outpatient care	Karolinska Inst	Diagnoses
Conscript register	Pliktverket	Test results

Table 3: Swedish Registers

Data collection from national registers, along with genetic data, will be important contributions to the study of the etiology, consequences and long-term outcome of the prevalent but understudied anxiety disorders. As mentioned previously, we are aware of the relatively small sample size of this study in order to analyze genetic and environmental risk factors for development of an anxiety disorder. However, in a long-term perspective this material will be part of a bigger effort at our clinic to build a sufficiently large material in order to answer research questions related to these matters.

Intervention

ICBT

The guided ICBT treatment program is an internet-based intervention that will involve youths as well as their parents. The program is founded on and inspired by previously evaluated and evidence-based interventions [e.g., 66, 67] and has been used in a previous trial by our research group. Altogether, youth go through 10 chapters/sessions. The program is divided into three different phases, starting

with psychoeducation regarding social anxiety and the rationale for a cognitive behavioral intervention. Phase two is the main part of treatment and contains cognitive and behavioral interventions, mainly exposure and habituation to feared situations and/or stimuli. During exposure exercises, the participant confronts him-/herself with situations that normally trigger anxiety and learns how to remain present in these situations (i.e., to not avoid or escape or use safety-behaviors) in order to habituate to anxiety. Phase three addresses maintenance of treatment gains and relapse prevention. The chapters consist of texts to read, films and illustrations to watch as well as different kinds of exercises for the participants to do. During the 10-week intervention the participants will also have contact with their therapist over the telephone or over Skype. This direct communication may target challenging aspects of the treatment, such as constructing an idiosyncratic exposure-hierarchy, as well as giving the participant the opportunity to perform exposure-exercises together with the therapist.

Control condition

The control condition is internet-delivered support and counseling consisting of 10 chapters. The counseling chapters consists of information about social anxiety and questions regarding how the participants have experienced their social anxiety the last week and how they have handled their problems. Information will also be given about life style factors such as sleep and nutrition. No active CBT-components will be included in the program.

Parents

Parents will go through a shorter program (5 internet-delivered chapters) in both conditions. The parent chapters in ICBT contain psychoeducative material and information/exercises about family accommodation, parental coping strategies and how to coach the youth through treatment. The parent chapters in the control condition contain the same information as the youths'. Parents and youths will have separate login accounts to the internet-based platform.

During the treatment phase (10 weeks), regardless of treatment condition, participants (youth as well as parents) will have regular contact with a therapist through e-mails, phone calls/Skype and through standardized forms in the program.

Statistical analysis

Outcomes will be described as significant changes in clinician, self- and parent-rated measures of anxiety symptoms and comorbid symptoms, within- and between group effect sizes (Cohen's *d*), clinically significant improvement rates and remission rates. Analyses will involve t-tests as well as linear mixed-effects modeling. Randomness of missing data will be analyzed with logistic regression. Depending on the amount of missing data, multiple imputations will be employed to compensate for missing values.

PROJECT SCHEDULE

Autumn 2017-Spring 2018: Recruitment and inclusion of participants.

Autumn 2017 – Autumn 2018: Treatment of *N* = 101 youths with SAD.

Autumn 2018: Analysis of data. Presentation and publication of pre-post results.

Spring 2018 - autumn 2019: 3- and 12-month follow-up.

ANTICIPATED EFFECTS

The overall goal of our research is to develop an effective treatment for youth SAD. This study contributes with information about efficacy of therapist-guided ICBT treatment. In the long run we believe that therapist-guided ICBT could have the potential to increase the availability of treatment of SAD in youth, both in a geographical and temporal sense. This would be desirable not only for patients and their families, but also for health care establishments, who could decrease their waiting times. Moreover, internet-delivered interventions could potentially supply effective treatment at a lower cost, compared with traditional face-to-face CBT.

ETHICAL CONSIDERATIONS

ICBT differs from traditional therapy in the amount of information that the therapist is provided with. A thorough baseline assessment decreases the risk that patients in need of more extensive care are included in the study. Close monitoring of symptoms and the severity of symptoms throughout the treatment (continuous self- and parent ratings) ensures that families with complex and/or worsened problems can be detected and referred to more suitable treatments. The e-mail and telephone/Skype communication, ensures that families with problems too severe for this program can be identified and referred to more suitable treatments. The participants may worry about computer safety and maintenance of confidentiality. The families are provided with information about the risks and precautions that are being taken when using communication technology, e.g., encrypted server technology and double authentication with SMS-logins. International rules and regulations will be followed. In our view, the ethical risk is limited since every study participant will receive treatment.

PRESENTATION OF RESULTS

The study results will be presented in three articles submitted for publication in scientific journals:

1. Internet-delivered CBT versus internet-delivered support and counseling for youths with social anxiety disorder: a randomized controlled trial
2. Mechanisms of change in ICBT for youths with SAD
3. Predictors of outcome and long-term effects of Internet-delivered CBT

Eventually, results from analyses of the DNA material collected within this study (along with samples collected in future studies from this research group and from studies conducted by collaborating research groups) will be submitted for publication in scientific journals.

Data collected on self- and parent measures may be used for psychometric validation of the scales.

The results will also be presented at international and national scientific conferences. Part of the collected data may also be used in a master thesis written by students at the psychologist program at Karolinska Institutet.

References

1. Association, A.P., *Diagnostic and statistical manual of mental disorders (DSM-5®)*. 2013: American Psychiatric Pub.

2. Lawrence, D., et al., *The Mental Health of Children and Adolescents: Report on the Second Australian Child and Adolescent Survey of Mental Health and Wellbeing.* 2015.

3. Burstein, M., et al., *Social phobia and subtypes in the national comorbidity survey-adolescent supplement: prevalence, correlates, and comorbidity.* J Am Acad Child Adolesc Psychiatry, 2011. **50**(9): p. 870-80.

4. Beesdo, K., et al., *Incidence of social anxiety disorder and the consistent risk for secondary depression in the first three decades of life.* Archives of General Psychiatry, 2007. **64**(8): p. 903-912.

5. Buckner, J.D., et al., *Specificity of social anxiety disorder as a risk factor for alcohol and cannabis dependence.* Journal of psychiatric research, 2008. **42**(3): p. 230-239.

6. Katzelnick, D.J., et al., *Impact of generalized social anxiety disorder in managed care.* American Journal of Psychiatry, 2001. **158**(12): p. 1999-2007.

7. Acarturk, C., et al., *Economic costs of social phobia: a population-based study.* Journal of affective disorders, 2009. **115**(3): p. 421-429.

8. Patel, A., et al., *The economic consequences of social phobia.* Journal of affective disorders, 2002. **68**(2): p. 221-233.

9. Hofmann, S.G. and J.A. Smits, *Cognitive-behavioral therapy for adult anxiety disorders: a meta-analysis of randomized placebo-controlled trials.* J Clin Psychiatry 2008. **69**(4): p. 621.

10. Scaini, S., et al., *A comprehensive meta-analysis of cognitive-behavioral interventions for social anxiety disorder in children and adolescents.* J Anxiety Disord, 2016. **42**: p. 105-112.

11. Segool, N.K. and J.S. Carlson, *Efficacy of cognitive ‐ behavioral and pharmacological treatments for children with social anxiety.* Depression and Anxiety, 2008. **25**(7): p. 620-631.

12. von Knorring, L., et al., *SBU utvärderar sjukvårdens metoder.* 2005.

13. Pilling, S., et al., *Recognition, assessment and treatment of social anxiety disorder: summary of NICE guidance.* Bmj, 2013. **346**: p. f2541.

14. Rapee, R.M., C.A. Schniering, and J.L. Hudson, *Anxiety disorders during childhood and adolescence: Origins and treatment.* Annual review of clinical psychology, 2009. **5**: p. 311-341.

15. Essau, C.A., J. Conradt, and F. Petermann, *Frequency and comorbidity of social phobia and social fears in adolescents.* Behav Res Ther, 1999. **37**(9): p. 831-43.

16. Wittchen, H.U., M.B. Stein, and R.C. Kessler, *Social fears and social phobia in a community sample of adolescents and young adults: prevalence, risk factors and co-morbidity.* Psychological Medicine, 1999. **29**(2): p. 309-323.

17. Merikangas, K.R., et al., *Service utilization for lifetime mental disorders in US adolescents: Results of the National Comorbidity Survey–Adolescent Supplement (NCS-A).* Journal of the American Academy of Child & Adolescent Psychiatry, 2011. **50**(1): p. 32-45.

18. Hedman, E., B. Ljótsson, and N. Lindefors, *Cognitive behavior therapy via the Internet: a systematic review of applications, clinical efficacy and cost–effectiveness.* Expert review of pharmacoeconomics & outcomes research, 2012. **12**(6): p. 745-764.

19. Hedman, E., et al., *Internet-based cognitive behavior therapy vs. cognitive behavioral group therapy for social anxiety disorder: a randomized controlled non-inferiority trial.* PLoS One, 2011. **6**(3): p. e18001.

20. March, S., S.H. Spence, and C.L. Donovan, *The efficacy of an internet-based cognitive-behavioral therapy intervention for child anxiety disorders.* Journal of pediatric psychology, 2009. **34**(5): p. 474-487.

21. Tillfors, M., et al., *A randomized trial of Internet-delivered treatment for social anxiety disorder in high school students.* Cogn Behav Ther, 2011. **40**(2): p. 147-57.

22. Donovan, C.L. and S. March, *Online CBT for preschool anxiety disorders: a randomised control trial.* Behaviour research and therapy, 2014. **58**: p. 24-35.

23. Spence, S.H., et al., *A randomized controlled trial of online versus clinic-based CBT for adolescent anxiety.* J Consult Clin Psychol, 2011. **79**(5): p. 629-42.

24. Spence, S.H., et al., *Generic versus disorder specific cognitive behavior therapy for social anxiety disorder in youth: A randomized controlled trial using internet delivery.* Behav Res Ther, 2017. **90**: p. 41-57.

25. Nordh, M., et al., *Therapist-guided Internet-delivered cognitive behavior therapy supplemented by group-exposure sessions for adolescents with Social Anxiety Disorder: a feasibility trial* Manuscript.

26. Eley, T.C., et al., *Therapygenetics: the 5HTTLPR and response to psychological therapy.* Molecular psychiatry, 2012. **17**(3): p. 236.

27. Belsky, J. and M. Pluess, *Beyond diathesis stress: differential susceptibility to environmental influences.* Psychological bulletin, 2009. **135**(6): p. 885.

28. APA, *Diagnostic and statistical manual of mental disorders*. Fifth ed. 2013, Arlington, VA: American Psychiatric Association.

29. Silverman, W.K. and A.M. Albano, *Anxiety Disorders Interview Schedule for DSM-IV: Parent interview schedule*. 1996, Albany, NY: Greywind Publications.

30. Silverman, W.K., L.M. Saavedra, and A.A. Pina, *Test-retest reliability of anxiety symptoms and diagnoses with the anxiety disorders interview schedule for DSM-IV: Child and parent versions.* Journal of the American Academy of Child and Adolescent Psychiatry, 2001. **40**(8): p. 937-944.

31. Wood, J.J., et al., *Concurrent validity of the anxiety disorders section of the anxiety disorders interview schedule for DSM-IV: Child and parent versions.* Journal of Clinical Child and Adolescent Psychology, 2002. **31**(3): p. 335-342.

32. Guy, W., *Assessment manual for psychopharmacology, revised*. 1976, Washington DC: US Government Printing Office.

33. Shaffer, D., et al., *A children's global assessment scale (CGAS).* Archives of General psychiatry, 1983. **40**(11): p. 1228-1231.

34. Bird, H.R., et al., *Further measures of the psychometric properties of the Children's Global Assessment Scale.* Archives of General Psychiatry, 1987. **44**(9): p. 821-824.

35. Lundh, A., et al., *Children's Global Assessment Scale (CGAS) in a naturalistic clinical setting: Inter-rater reliability and comparison with expert ratings.* Psychiatry research, 2010. **177**(1-2): p. 206-210.

36. Simpson, H.B., et al., *Development of a Patient Adherence Scale for Exposure and Response Prevention Therapy.* Behavior Therapy, 2010. **41**(1): p. 30-37.

37. Masia-Warner, C., et al., *The Liebowitz social anxiety scale for children and adolescents: an initial psychometric investigation.* J Am Acad Child Adolesc Psychiatry, 2003. **42**(9): p. 1076-84.

38. Storch, E.A., et al., *Factor structure of the Liebowitz Social Anxiety Scale for Children and Adolescents.* Child Psychiatry Hum Dev, 2006. **37**(1): p. 25-37.

39. Olivares, J., R. Sanchez-Garcia, and J.A. Lopez-Pina, *The Liebowitz Social Anxiety Scale for Children and Adolescents.* Psicothema, 2009. **21**(3): p. 486-91.

40. Schmits, E., A. Heeren, and E. Quertemont, *The self-report version of the LSAS-CA: Psychometric properties of the French version in a non-clinical adolescent sample.* Psychologica Belgica, 2014. **54**(2).

41. Chorpita, B.F., et al., *Assessment of symptoms of DSM-IV anxiety and depression in children: A revised child anxiety and depression scale.* Behaviour research and therapy, 2000. **38**(8): p. 835-855.

42. Chorpita, B.F., C.E. Moffitt, and J. Gray, *Psychometric properties of the Revised Child Anxiety and Depression Scale in a clinical sample.* Behaviour research and therapy, 2005. **43**(3): p. 309-322.

43. Ebesutani, C., et al., *A psychometric analysis of the revised child anxiety and depression scale—parent version in a clinical sample.* Journal of abnormal child psychology, 2010. **38**(2): p. 249-260.

44. Hinrichsen, H. and D.M. Clark, *Anticipatory processing in social anxiety: Two pilot studies.* Journal of behavior therapy and experimental psychiatry, 2003. **34**(3): p. 205-218.

45. Clark, D.M., et al., *Social behaviour questionnaire.* Unpublished questionnaire., 1995.

46. McEvoy, P.M. and P. Kingsep, *The post-event processing questionnaire in a clinical sample with social phobia.* Behaviour Research and Therapy, 2006. **44**(11): p. 1689-1697.

47. Rachman, S., J. Grüter-Andrew, and R. Shafran, *Post-event processing in social anxiety.* Behaviour Research and Therapy, 2000. **38**(6): p. 611-617.

48. Woody, S.R., *Effects of focus of attention on anxiety levels and social performance of individuals with social phobia.* J Abnorm Psychol, 1996. **105**(1): p. 61-9.

49. Hodson, K.J., et al., *Can Clark and Wells'(1995) cognitive model of social phobia be applied to young people?* Behavioural and Cognitive Psychotherapy, 2008. **36**(04): p. 449-461.

50. Miers, A., et al., *Subjective and objective arousal correspondence and the role of self-monitoring processes in high and low socially anxious youth.* Journal of Experimental Psychopathology, 2011. **2**(4): p. 531-550.

51. Cuming, S., et al., *A self-report measure of subtle avoidance and safety behaviors relevant to social anxiety: development and psychometric properties.* J Anxiety Disord, 2009. **23**(7): p. 879-83.

52. Thomas, S.A., et al. *Using the Subtle Avoidance Frequency Examination in adolescent social anxiety assessments*. in *Child & Youth Care Forum*. 2012. Springer.

53. Stevens, K., *Assessing the performance of a new generic measure of health-related quality of life for children and refining it for use in health state valuation.* Appl Health Econ Health Policy, 2011. **9**(3): p. 157-69.

54. Attkisson, C.C. and R. Zwick, *The Client Satisfaction Questionnaire: Psychometric properties and correlations with service utilization and psychotherapy outcome.* Evaluation and program planning, 1982. **5**(3): p. 233-237.

55. Borkovec, T.D. and S.D. Nau, *Credibility of analogue therapy rationales.* Journal of Behavior Therapy and Experimental Psychiatry, 1972. **3**(4): p. 257-260.

56. Mundt, J.C., et al., *The Work and Social Adjustment Scale: a simple measure of impairment in functioning.* British Journal of Psychiatry, 2002. **180**: p. 461-464.

57. Zigmond, A.S. and R.P. Snaith, *The hospital anxiety and depression scale.* Acta psychiatrica scandinavica, 1983. **67**(6): p. 361-370.

58. Andersson, G., et al., *Internet administration of the Hospital Anxiety and Depression Scale in a sample of tinnitus patients.* Journal of Psychosomatic Research, 2003. **55**(3): p. 259-262.

59. Bjelland, I., et al., *The validity of the Hospital Anxiety and Depression Scale - An updated literature review.* Journal of Psychosomatic Research, 2002. **52**(2): p. 69-77.

60. Saunders, J.B., et al., *Development of the Alcohol Use Disorders Identification Test (AUDIT): WHO Collaborative Project on Early Detection of Persons with Harmful Alcohol Consumption--II.* Addiction, 1993. **88**(6): p. 791-804.

61. Berman, A.H., et al., *Evaluation of the Drug Use Disorders Identification Test (DUDIT) in criminal justice and detoxification settings and in a Swedish population sample.* Eur Addict Res, 2005. **11**(1): p. 22-31.

62. Lebowitz, E.R., et al., *Family accommodation in pediatric anxiety disorders.* Depress Anxiety, 2013. **30**(1): p. 47-54.

63. Kagan, E.R., et al., *Accomodation and treatment of anxious youth.* Depress Anxiety, 2016. **33**(9): p. 840-7.

64. Bouwmans, C., et al., *Feasibility, reliability and validity of a questionnaire on healthcare consumption and productivity loss in patients with a psychiatric disorder (TiC-P).* BMC Health Services Research, 2013. **13**.

65. Rozental, A., et al., *Negative Effects of Psychological Treatments: An Exploratory Factor Analysis of the Negative Effects Questionnaire for Monitoring and Reporting Adverse and Unwanted Events.* PLoS One, 2016. **11**(6): p. e0157503.

66. Albano, A.M. and P.M. DiBartolo, *Cognitive-Behavioral Therapy for Social Phobia in Adolescents: Stand Up, Speak Out Therapist Guide*. 2007: Oxford University Press.

67. Beidel, D.C., S.M. Turner, and T.L. Morris, *Behavioral treatment of childhood social phobia.* Journal of consulting and clinical psychology, 2000. **68**(6): p. 1072.
