# Supplementary material for: Therapist-Guided Internet-Delivered Cognitive Behavioral Therapy vs Internet-Delivered Supportive Therapy for Children and Adolescents With Social Anxiety Disorder: A Randomized Clinical Trial
Source: JAMA Psychiatry. 2021 May 12;78(7):1–9. doi: 10.1001/jamapsychiatry.2021.0469 (PMC8117054; doi:10.1001/jamapsychiatry.2021.0469)
Supplement: Supplement 2. — eMethods eResults. eReferences eTable 1. List of Measures eTable 2. Overview of Assessment Points eTable 3. Treatment Content for Ten Weeks of ICBT eTable 4. Treatment Content for Ten Weeks of ISUPPORT eTable 5. Treatment Completion, Credibility, Satisfaction and Adherence as Well as Therapist Time eTable 6. Observed Means, Standard Deviations and Model-Implied Standardized Mean Difference (Effect Size d) of Primary and Secondary Outcomes at Post Treatment eTable 7. Secondary Outcomes for Participants Receiving ICBT 3-Months After ISUPPORT. Analysis Includes Complete Cases eTable 8. Unit Costs and Sources eTable 9. Resource Use Frequencies eFigure 1. Cost-effectiveness Plane Regarding Societal Cost and QALYs (Quality Adjusted Life Years) eFigure 2. Willingness-to-Pay Graph Depicting the Probability of ICBT to Be Cost-effective Compared to ISUPPORT: Societal Perspective (Total Costs and Diagnostic Status) eFigure 3. Willingness-to-Pay Graph Depicting the Probability of ICBT to Be Cost-effective Compared to ISUPPORT. Health Care Provider Perspective (Treatment Costs and Diagnostic Status) eFigure 4. Sub-total Cost Differences Between ICBT and ISUPPORT During the Period From Baseline to 3-Month Follow-up. *p < 0.05 [file jamapsychiatry-e210469-s002.pdf]

Supplementary Online Content

Nordh M, Wahlund T, Jolstedt M, et al. Therapist-guided internet-delivered cognitive behavioral therapy vs internet-delivered supportive therapy for children and adolescents with social anxiety disorder: a randomized clinical trial. *JAMA Psychiatry*. Published online May 12, 2021. doi:10.1001/jamapsychiatry.2021.0469

eMethods

eResults

References

eTable 1. List of measures

eTable 2. Overview of assessment points

eTable 3. Treatment content for ten weeks of ICBT

eTable 4. Treatment content for ten weeks of ISUPPORT

eTable 5. Treatment completion, credibility, satisfaction and adherence as well as therapist time

eTable 6. Observed means, standard deviations and model-implied standardized mean difference (effect size d) of primary and secondary outcomes at post treatment

eTable 7. Secondary outcomes for participants receiving ICBT 3-months after ISUPPORT. Analysis includes complete cases

eTable 8. Unit costs and sources

eTable 9. Resource use frequencies

eFigure 1. Cost-effectiveness plane regarding societal cost and QALYs (quality adjusted life years)

eFigure 2. Willingness-to-pay graph depicting the probability of ICBT to be cost-effective compared to ISUPPORT. Societal perspective (total costs and diagnostic status)

eFigure 3. Willingness-to-pay graph depicting the probability of ICBT to be cost-effective compared to ISUPPORT. Health care provider perspective (treatment costs and diagnostic status)

eFigure 4. Sub-total cost differences between ICBT and ISUPPORT during the period from baseline to 3-month follow-up. *p < 0.05

This supplementary material has been provided by the authors to give readers additional information about their work.

© 2021 Nordh M et al. *JAMA Psychiatry.*

eMethods

Trial monitoring

An external observer from Karolinska Trial Alliance, an independent clinical trials unit, monitored the trial regularly to check that ethical and scientific quality standards were consistently high and in line with Good Clinical Practice (GCP).[1] This included monitoring that written consent was collected from all participants, that all inclusion and no exclusion criteria were fulfilled, that the data from the primary outcome measure were entered correctly into the database and that any adverse event was recorded, handled and reported correctly.

Outcomes

The ADIS was administered at the clinic (pre-treatment and 3-month follow-up) or over the phone (post-treatment). Secondary outcomes were the proportion of participants no longer fulfilling diagnostic criteria for SAD and the following measures: Improvement relative to pre-treatment was measured with the masked assessor-rated Clinical Global Impression – Improvement (CGI-I).[2] The Children's Global Assessment Scale (CGAS)[3] assessed global functioning (0-100) in youth. The internet intervention Patient Adherence Scale (iiPAS)[4] rated participants' adherence to various aspects of the treatment (work pace, engagement, communication with therapist, motivation to change and login frequency). Child- and parent-reported social anxiety was measured with the Liebowitz Social Anxiety Scale for Children and Adolescents (LSAS-CA).[5] Comorbid parent-reported anxiety and depression, as well as child-reported depressive symptoms were measured with the Revised Children's Anxiety and Depression Scale – Parent version (RCADS-P) and the Revised Children's Anxiety and Depression Scale – Depression subscale - Child version (RCADS-dep-C), respectively.[6] Quality of life was measured with the child-reported Child Health Utility 9D (CHU9D).[7] Treatment credibility and satisfaction were measured with the Treatment credibility and expectancy – child and parent version (C-scale)[8] and the Client Satisfaction Questionnaire – child and parent version (CSQ-C/P)[9], respectively. The parent version of Work and Social Adjustment Scale (WSAS-P)[10] was used to measure general functioning.

The Trimbos/iMTA questionnaire for Costs associated with Psychiatric Illness – Parent version (TIC-P) measured health-related costs.[11] During treatment, youths and parents were continuously asked to report any adverse events, which were also captured with the symptom subscale of the Negative Effects Questionnaire - Child Version (NEQ)[12] at post-treatment.

Parents also responded to demographic questions (e.g., parental education level and occupational status, birth country of the child, prior treatment history, and duration of social anxiety symptoms).

Therapists

All supporting therapists (*N*=7) were clinical psychologists who met weekly to discuss the progress of participants in the two interventions in order to strengthen treatment fidelity and participant safety. All therapists provided support to participants in both of the treatment arms and had at least one year of training in CBT (range 1-10 years; *M*=5.9 *SD*=4.0). Specific experience from working with socially anxious youths ranged from 0-7 years (*M*=2.6, *SD*=2.6).

Therapist time was measured as the total time therapists spent supporting the youth and the parent over the 10 weeks of treatment, including time spent giving written feedback online (automatically logged within the treatment platform) and time spent on the video call sessions (registered by the therapists).

Parental involvement

Parents of 10-14-year olds were instructed to help the child with the online material and to participate during the video call sessions, whereas parents of 15-17-year olds were instructed to let their adolescent decide the level of parental involvement throughout the treatment. Parents completed the Hospital Anxiety and Depression Scale (HADS)[13] to report their own level of anxiety and depression at pre-treatment.

Alcohol and substance use screening

The Alcohol Disorder Identification Test (AUDIT)[14] and the Drug Use Disorders Identification Test (DUDIT)[15] were administered to screen for alcohol- or drug abuse at pre-treatment among the youths.

Interventions

The two interventions were provided within a secure online platform and had similar structures and features: asynchronous therapist support (weekly through a built in message function, comments on work sheets and, if necessary, phone calls), treatment dose (10 consecutive online modules provided as one module per week over 10 weeks), video calls (three 20-30 minute sessions provided at weeks 3, 5 and 7) and format of online material (texts, films and audio clips, as well as interactive exercises and work sheets). Both treatments also

included general information about SAD, such as prevalence and etiology, as well as a weekly assignment to monitor fluctuations in the severity of SAD symptoms.

While the content of the comparator is not identical to the concept of "supportive therapy", as defined by the American Psychiatric Association (APA), there are more similarities than differences.

Cross-over cases
For participants crossing over from ISUPPORT to ICBT (three months after the termination of ISUPPORT), the ADIS diagnostic interview as well as child- and parent-report measures were administered after ICBT and at the 3-month follow-up after ICBT. However, the assessors were no longer masked to treatment allocation during this phase of the trial.

Reliability measures
Experienced instructors (MN and JH) trained all study assessors, to strengthen the reliability of the ADIS diagnostic assessments. All pre-treatment diagnostic interviews were video recorded and assessors watched and rated between three to five videos and discussed these with the instructors until agreement was reached. At the end of the trial, an independent assessor, otherwise not involved in the trial or the research group, rated a randomly selected 20% of the video recordings in order to estimate the inter-rater reliability of participants' SAD diagnoses. Masked assessors and therapists rated a random subset of video recordings (n=5) to estimate the intra-class correlation (ICC) for the CSR at pre-treatment. All video call sessions (see description below) were audio recorded and two independent assessors rated treatment fidelity in 20% of a random selection of recordings. Treatment fidelity among therapists was measured using a list of components from the two treatments. Raters noted the presence or absence of key components in each recording; treatment fidelity was considered good when the active CBT components were provided in the correct condition (e.g., exposure in ICBT), while none of these components were provided in the alternative condition (e.g., exposure in ISUPPORT).

Masking integrity
Participants were explicitly instructed not to disclose any information about their treatment allocation to the masked assessor in order to maintain the masking integrity. Masked assessors were six clinical psychologists (all with PhD degrees). We formally checked the integrity of the masked ratings. Specifically, masked assessors were asked to guess the participants' group allocation after each assessment. Assessors were also asked to choose the reason for their guess: 1) the family revealed the condition, 2) the amount of SAD-symptom reduction, or 3) a pure guess.[16]

Statistical analysis
Linear mixed effects models for continuous variables were fitted with full maximum likelihood estimation using the lme4 package[17] in R.[18] The best-fitted model was determined analytically for each outcome, and model selection was performed by means of likelihood ratio tests for nested models. All final models included fixed terms for treatment group (ICBT = 0.5, ISUPPORT = -0.5), time (coded in months from baseline) and a time by treatment interaction effects. Visual inspections of observed means for each treatment group and individual trajectories suggested a non-linear change over time. To linearize the relationship of the observed scores over time and to simplify the models with a single time trend, we chose a square root transformation of time, as suggested by Hedeker and Gibbons.[19] Correlated random effects were included if they significantly contributed to the model by means of likelihood ratio tests. Satterthwaite's approximation for denominator degrees of freedom was used for inferential tests. Effect sizes in the form of standardized mean difference (d) with confidence intervals were computed based on formulas provided by Feingold[20] (but here based on model-implied variance). Data were checked for violations to model assumptions (e.g., conditional normality, independence, homoscedasticity). Given some identified problems with influential observations and normality for certain variables (e.g., RCADS-P), we also reran a number of models with a robust linear mixed effects model using robustlmm package[21] in R, according to recommendations.[22] We also performed sensitivity analyses of inferential tests of key parameters in the model by computing 95% confidence intervals based on 3000 parametric bootstrap samples (using the 2.5th and 97.5th percentiles of parameter estimates from the empirical bootstrap distribution). While these sensitivity analyses did alter the results slightly, they did not alter the overall conclusions qualitatively. The logistic regression analysis for the secondary outcome variable SAD diagnosis at 3-month follow-up was fitted with full maximum likelihood estimation with non-normality robust standard errors using Mplus[23] (MLR estimation). The model included a binary predictor for treatment group (ICBT = 1, ISUPPORT = 0) and a continuous predictor for baseline severity ratings of CSR.

Maximum likelihood (ML) was used to handle missing data.[24] ML is one of two suggested techniques for analyzing data with missing information[25] because it provides unbiased estimates and standard errors under a more lenient missing data assumption (i.e., missing at random, MAR) than other commonly used methods.[24] Given the small amount of missing data and because no examined clinical or socio-demographic

© 2021 Nordh M et al. *JAMA Psychiatry.*

variables at baseline were significantly associated with the propensity for missing data at the post treatment assessment or the primary endpoint, we relied on ML under the MAR assumption for all primary outcome analyses.

Inter-rater reliability was measured using a kappa coefficient for presence of SAD-diagnosis.

Due to skewness of the cost data, missing data were handled with a non-parametric method, K-nearest neighbor imputation, using the DMwR package in R. Cost-estimations were carried out using generalized linear models (GLM) regression with gamma family distribution and log link, according to recommendations by Mihaylova and colleagues.[26] Costs were not discounted, as the time frame was less than one year. As sensitivity analyses, we provide cost-effectiveness planes, displaying the uncertainty of the analyses using 5000 bootstrapped cost and effect difference pairs as well as cost-effectiveness acceptability curves, displaying the probability of cost-effectiveness over a range of willingness-to-pay scenarios.

eResults

Masked assessor-rated improvement
At the 3-month follow-up, 35% were rated as *Much* or *Very much* improved (CGI-I≤2) in the ICBT group, compared to 20% in the ISUPPORT group (for complete cases), a statistically non-significant difference (χ^2(1, *N*=99)=2.69, *p*=.10).

Adherence, credibility and treatment satisfaction
There were no statistically significant between-group differences regarding treatment completion, overall therapist time spent on each family per week, or in therapists' ratings of participants' adherence (eTable 5 below). ICBT was rated as more credible and was associated with higher satisfaction than ISUPPORT (eTable 5 below). However, post-hoc analyses, based on complete cases, showed that credibility was not significantly correlated with diagnostic status at primary endpoint (r_{pb}(97)=0.13, *p*=.21), number of completed modules (*r*(99)=0.12, *p*=.25), therapist time (*r*(99)=0.18, *p*=.08) or change in CSR (*r*(99)=0.20, *p*=.05). Similarly, there was no significant association between treatment satisfaction and number of completed modules (*r*(84)=0.13, *p*=.24) or therapist time (*r*(84)=0.09, *p*=.44). Satisfaction had a small but significant association with diagnostic status at primary endpoint (r_{pb}(83)=-0.31, *p*=.004) and change in CSR (*r*(83)=0.37, *p*=.001).

Masking integrity

The careful masking procedures during the diagnostic interviews resulted in only three families accidentally revealing the treatment allocation (ICBT in all three cases). However, none of these cases were assessed to be free from the SAD diagnosis at the 3-month follow-up, nor were they assessed to be much or very much improved (CGI-I<3), limiting the risk that the effect of ICBT was inflated. Excluding these three families, masked assessors guessed the right treatment allocation in 63% of the cases at primary endpoint (χ^2(1)=6.57, *p*<.05), meaning that they were significantly more often correct than expected by chance (50%). However, the majority (57%) were pure guesses according to the assessors and the rest (43%) were guesses based on the participant's reduction of SAD symptoms. Furthermore, there was no significant difference in SAD severity (CSR) at the primary endpoint between participants whose group allocation was guessed correctly compared to those whose allocation was guessed incorrectly (*t*(96)=0.12, *p*=.91).

Post-hoc analyses conducted separately for the ICBT group and the ISUPPORT group regarding the masking integrity showed that participants whose true allocation to ICBT were guessed correctly by the assessor received lower CSR ratings (*m*=3.54, *sd*=1.46) than participants whose allocation were guessed wrongly (*m*=5.00, *sd*=0.78), a statistically significant difference, *t*(47)=3.52, *p*<.001. Conversely, among participants whose true allocation was ISUPPORT, those whose allocation was guessed correctly by the assessor received higher CSR ratings (*m*=5.07, *sd*=1.05) than those whose allocation were guessed wrongly (*m*=3.76, *sd*=1.22), also a statistically significant difference, *t*(47)=4.03, *p*<.001. This might indicate that the masked assessors had a preconception that ICBT would be more effective than ISUPPORT and that they therefore were more prone to guess that a participant had received ICBT if the participant reported symptom reductions during the assessment.

Cross-over cases
Thirty participants out of 52 (58%) who still met diagnostic criteria for SAD accepted the offer to start ICBT three months after completing ISUPPORT. A post-hoc analysis of the outcomes in this group (*n*=30) showed that there was a significant decrease in therapist rated SAD severity (CSR) from pre to post ICBT (*t*(26)=2.94, *p*<.01) and this improvement remained from post-treatment to the 3-month follow-up (*t*(24)=0.55, *p*=.59). The proportion of participants no longer fulfilling criteria for SAD at the 3-month follow-up was 27% (*n*=8). Outcomes for this group are presented in eTable 7 below.

© 2021 Nordh M et al. *JAMA Psychiatry.*

Adverse effects
Results from the NEQ-C (symptoms subscale) showed that 20 participants (39%) in ICBT and 15 participants (29%) in ISUPPORT reported having experienced at least some negative effect related to the treatment, such as disturbed sleep or increased anxiety. In addition, five (10%) participants in ICBT and two (4%) participants in ISUPPORT, reported an increase of conflicts with the parents due to the treatment. Suicidal ideation during the course of the treatment was reported by four participants in ICBT (8%) and six participants in ISUPPORT (12%). Statistical comparisons of above-mentioned proportions in the two groups were all none-significant (p>0.05).

The suicide attempt in ISUPPORT was managed in line with the predefined standard operating procedures (SOP) for serious adverse events, after which the family resumed participation in the trial. The SOP instructed therapists to immediately contact the caregiver, as well as to inform the trial coordinator (first author, MN), whenever they were informed about a serious adverse event such as suicidal intent, a suicide attempt or self-injury. Caregivers were contacted by telephone to make a safety plan and for therapists to be able to make an informed decision about appropriate care (i.e., if the family should continue participation in the trial or be referred to other mental health services). The therapist would also conduct a suicide risk assessment with the young person according to a predefined protocol. The safety plan, suicide risk assessment and treatment planning were documented in the participant's medical record.

Cost-effectiveness
Due to minimal differences in QALYs between ICBT and ISUPPORT (β=-0.011, t=-0.55, p=.58) it was not meaningful to calculate an ICER value regarding quality of life, in order to avoid an inflated ICER due to a near zero denominator in the equation. Seen from a health care provider perspective, the mean cost of ICBT was €176.84 (95% CI [148.74, 204.94]) and the mean cost of ISUPPORT was €145.36 (95% CI [124.96, 165.75]). Using the intervention cost of ICBT compared to ISUPPORT in relation to the rates of participants being free from SAD following the two interventions, the ICER was M=562.14 (95% CI [509.78, 614.50]), indicating an additional cost that is associated with higher probability of being free from SAD in the ICBT group. Again, due to near-zero differences, it was not meaningful to calculate an ICER value for QALYs relative to health care provider costs. Cost-effectiveness planes for QALYs and cost-effectiveness acceptability curves and sub-total cost differences between ICBT and ISUPPORT are provided in eFigures 1-4, as well as detailed cost analysis information (eTable 8 and 9).

The cost of school productivity loss is calculated as the number of days that the child attends school with a reduced ability to perform, multiplied by the average ability to perform on such days (reported by the parent in TIC-P on a 10-point Likert-scale from 0=no ability to perform to 10=normal ability to perform, which is transformed to percentage where 0=100% ability loss and 10=0% ability loss), and multiplied by the unit cost of one school day (based on cost of tuition in Sweden, see Presenteeism in eTable 8).

© 2021 Nordh M et al. *JAMA Psychiatry.*

References

1. Food and Drug Administration. *Consolidated Standards of Reporting Trials* 1996.
2. Guy W. *Assessment manual for psychopharmacology, revised.* Washington DC: US Government Printing Office; 1976.
3. Shaffer D, Gould MS, Brasic J, et al. A children's global assessment scale (CGAS). *Archives of general psychiatry.* 1983;40(11):1228-1231.
4. Lenhard F, Mitsell K, Jolstedt M, et al. The Internet Intervention Patient Adherence Scale for Guided Internet-Delivered Behavioral Interventions: Development and Psychometric Evaluation. *J Med Internet Res.* 2019.
5. Masia-Warner C, Storch EA, Pincus DB, Klein RG, Heimberg RG, Liebowitz MR. The Liebowitz social anxiety scale for children and adolescents: an initial psychometric investigation. *J Am Acad Child Adolesc Psychiatry.* 2003;42(9):1076-1084.
6. Chorpita BF, Yim L, Moffitt C, Umemoto LA, Francis SE. Assessment of symptoms of DSM-IV anxiety and depression in children: A revised child anxiety and depression scale. *Behav Res Ther.* 2000;38(8):835-855.
7. Stevens K. Assessing the performance of a new generic measure of health-related quality of life for children and refining it for use in health state valuation. *Applied health economics and health policy.* 2011;9(3):157-169.
8. Borkovec TD, Nau SD. Credibility of analogue therapy rationales. *Journal of Behavior Therapy and Experimental Psychiatry.* 1972;3(4):257-260.
9. Attkisson CC, Zwick R. The Client Satisfaction Questionnaire: Psychometric properties and correlations with service utilization and psychotherapy outcome. *Eval Program Plann.* 1982;5(3):233-237.
10. Jassi A, Lenhard F, Krebs G, et al. The work and social adjustment scale, youth and parent versions: psychometric evaluation of a brief measure of functional impairment in young people. 2019.
11. Bouwmans C, Jong KD, Timman R, et al. Feasibility, reliability and validity of a questionnaire on healthcare consumption and productivity loss in patients with a psychiatric disorder (TiC-P). *Bmc Health Serv Res.* 2013;13.
12. Rozental A, Kottorp A, Boettcher J, Andersson G, Carlbring P. Negative Effects of Psychological Treatments: An Exploratory Factor Analysis of the Negative Effects Questionnaire for Monitoring and Reporting Adverse and Unwanted Events. *PLoS One.* 2016;11(6):e0157503.
13. Zigmond AS, Snaith RP. The hospital anxiety and depression scale. *Acta Psychiat Scand.* 1983;67(6):361-370.
14. Saunders JB, Aasland OG, Babor TF, de la Fuente JR, Grant M. Development of the Alcohol Use Disorders Identification Test (AUDIT): WHO Collaborative Project on Early Detection of Persons with Harmful Alcohol Consumption--II. *Addiction.* 1993;88(6):791-804.
15. Berman AH, Bergman H, Palmstierna T, Schlyter F. Evaluation of the Drug Use Disorders Identification Test (DUDIT) in criminal justice and detoxification settings and in a Swedish population sample. *Eur Addict Res.* 2005;11(1):22-31.
16. Basoglu M, Marks I, Livanou M, Swinson R. Double-blindness procedures, rater blindness, and ratings of outcome: observations from a controlled trial. *Arch Gen Psychiatry.* 1997;54(8):744-748.
17. Bates D, Maechler M, Bolker B, Walker S. lme4: Linear mixed-effects models using Eigen and S4. R package version 1.1-7. In:2014.
18. Team RC. R: A language and environment for statistical computing. In: Vienna, Austria; 2016.
19. Hedeker D, Gibbons RD. *Longitudinal data analysis.* Vol 451: John Wiley & Sons; 2006.
20. Feingold A. Confidence interval estimation for standardized effect sizes in multilevel and latent growth modeling. *J Consult Clin Psychol.* 2015;83(1):157.

© 2021 Nordh M et al. *JAMA Psychiatry.*

21. Koller M. robustlmm: an R package for robust estimation of linear mixed-effects models. *Journal of statistical software.* 2016;75(6):1-24.
22. Field AP, Wilcox RR. Robust statistical methods: A primer for clinical psychology and experimental psychopathology researchers. *Behav Res Ther.* 2017;98:19-38.
23. Muthén L, Muthén B. Mplus user's guide. Eighth. *Muthen & Muthen.* 2017.
24. Enders CK. *Applied missing data analysis.* Guilford press; 2010.
25. Schafer JL, Graham JW. Missing data: our view of the state of the art. *Psychol Methods.* 2002;7(2):147.
26. Mihaylova B, Briggs A, O'Hagan A, Thompson SG. Review of statistical methods for analysing healthcare resources and costs. *Health Econ.* 2011;20(8):897-916.

© 2021 Nordh M et al. *JAMA Psychiatry.*

eTable 1: List of measures.

Category	Therapist/masked assessor-rated	Child self-report	Parent report
Diagnostic status/ comorbidity	ADIS-C		HADS
Anxiety symptom severity	CSR<br>CGI-I	LSAS-CA<br>RCADS-C-dep*	LSAS-P<br>RCADS-P
Alcohol- and substance abuse		AUDIT<br>DUDIT	
Impairment	CGAS		WSAS-P
Quality of life		CHU9D	
Cost-effectiveness			TiC-P
Adherence/ compliance	iiPAS		
Treatment satisfaction/<br>Treatment credibility		CSQ-C<br>C-scale-C	CSQ-P<br>C-scale-P
Adverse events		NEQ-C	NEQ-P

ADIS-C=Anxiety Disorder Interview Schedule for DSM-IV-Child version; HADS=Hospital Anxiety and Depression Scale; CSR=Clinician Severity Rating; LSAS-C/P=Liebowitz Social Anxiety Scale-Child/Parent version; CGI-I=Clinical Global Impression - Improvement; RCADS-C-dep=Revised Children's Anxiety and Depression Scale-Child version-Depression; RCADS-P= Revised Children's Anxiety and Depression Scale-Parent version; AUDIT=Alcohol Disorder Identification Test; DUDIT=Drug Use Disorders Identification Test; CGAS=Children's Global Assessment Scale; WSAS-P=Work and Social Adjustment Scale-Parent version; CHU9D=Child Health Utility 9D; TIC-P= Trimbos/iMTA questionnaire for Costs associated with Psychiatric Illness–Parent version; iiPAS=internet intervention Patient Adherence Scale; CSQ-C/P= Client Satisfaction Questionnaire–Child/Parent Version; C-scale-C/P= Treatment credibility and expectancy–Child/Parent version; NEQ-C/P=Negative Effects Questionnaire–Child/Parent Version

© 2021 Nordh M et al. *JAMA Psychiatry.*

eTable 2: Overview of assessment points.

	Telephone interview	Pre treatment	After 17 days	After 35 days	Post treatment	3-month follow-up
Therapist/ masked assessor-rated						
Incl./Excl.	X	X				
Demographic		X				
ADIS-C/P		X			X	X
CSR		X			X	X
CGI-I					X	X
CGAS		X			X	X
iiPAS				X	X	X
Child-/parent reported						
Demography		X				
LSAS-C/P		X			X	X
RCADS-C*/P		X*			X*	X*
HADS		X				
WSAS-P		X			X	X
TIC-P		X			X	X
CHU9D		X			X	X
C-scale-C/P			X			
CSQ-C/P					X	
AUDIT		X				
DUDIT		X				
NEQ-C/P					X	

ADIS-C=Anxiety Disorder Interview Schedule for DSM-IV-Child version; HADS=Hospital Anxiety and Depression Scale; CSR=Clinician Severity Rating; LSAS-C/P=Liebowitz Social Anxiety Scale-Child/Parent version; CGI-I=Clinical Global Impression - Improvement; RCADS-C-dep=Revised Children's Anxiety and Depression Scale-Child version-Depression; RCADS-P= Revised Children's Anxiety and Depression Scale-Parent version; AUDIT=Alcohol Disorder Identification Test; DUDIT=Drug Use Disorders Identification Test; CGAS=Children's Global Assessment Scale; WSAS-P=Work and Social Adjustment Scale-Parent version; CHU9D=Child Health Utility 9D; TIC-P=Trimbos/iMTA questionnaire for Costs associated with Psychiatric Illness–Parent version; iiPAS=internet intervention Patient Adherence Scale; CSQ-C/P= Client Satisfaction Questionnaire–Child/Parent Version; C-scale-C/P= Treatment credibility and expectancy–Child/Parent version; NEQ-C/P=Negative Effects Questionnaire–Child/Parent Version. *Only RCADS Depression subscale and suicidal ideation item was administered to children.

© 2021 Nordh M et al. *JAMA Psychiatry.*

eTable 3. Treatment content for ten weeks of ICBT.

	Child/adolescent		Parent
	Online material	Video call sessions	Online material
1	Introduction to ICBT. Fear response, social anxiety and self-focus. How to do functional analyses of one's own behaviour. Anxiety rating thermometer (0-100).		Introduction to ICBT. Fear response. Development and prognostic factors for SAD. Anxiety disorders. Functional analyses of the child's anxiety, and parental reactions.
2	Learn about safety behaviours and avoidance. Skills training: focus shifting. Social interaction with and without safety behaviours.		Safety behaviours. Maintaining factors. Goal setting. Exposure. Self-focus and focus shifting.
3	Learn about exposure. Goal setting and exposure hierarchy.	Exposure hierarchy. In vivo exposure.	Parental accommodation. Problem solving. Rewards and positive interaction.
4	Social skills training. Exposure follow-up and planning.		Exposure planning. Social skills. Parent management.
5	Thought traps and finding alternative/adaptive thoughts. Exposure follow-up and planning.	In vivo exposure.	Evaluation, summary and exposure planning.
6	Anticipatory anxiety and post-event processing. Focus shifting and exposure planning.		
7	Self-assertiveness training. Follow-up safety behaviours. Follow-up exposure and focus shifting.	In vivo exposure.	
8	Summary of treatment content. Exposure follow-up and planning.		
9	Social mishaps. Exposure follow-up and planning.		
10	Relapse prevention.		

© 2021 Nordh M et al. *JAMA Psychiatry.*

eTable 4. Treatment content for ten weeks of ISUPPORT.

	Child/adolescent		Parent
	Online material	Video call sessions	Online material
1	Introduction to ISUPPORT. Psychoeducation about social anxiety. Introduction to healthy habits.		Introduction to ISUPPORT. Fear response. Development and prognostic factors for SAD. Anxiety disorders.
2	Monitor SAD symptoms Learning about anxiety and fear response. Sleeping habits.		Monitor child's SAD-symptoms. Self-focus. Sleeping habits, eating and nutrition and physical activity.
3	Follow-up of sleeping habits. Etiology of SAD. Eating and nutrition.	Monitor SAD-symptoms. Encourage strategies to manage social anxiety.	Social fear in the brain. Parental challenges. Self-compassion.
4	Follow-up of eating and nutrition. Feared social situations. Learning about stress and recovery.		Learning about stress and recovery.
5	Follow-up of stress and recovery. Self-focus and blushing. The importance of physical activity.	Monitor SAD-symptoms. Encourage strategies to manage social anxiety and to use healthy habits to improve wellbeing.	Family relationships. Protective factors.
6	Follow-up of physical activity. Learning about protective factors and school performance.		
7	Learning about comorbid disorders. Family relationships.	Monitor SAD-symptoms. Encourage strategies to manage social anxiety, encourage healthy habits and activate social support. Guide self-reflection about association between SAD and comorbidity.	
8	Social fear in the brain. Friendships and love.		
9	Embarrassment and blushing. Self-esteem and self-confidence. Self-compassion.		
10	Summary of healthy habits and relationships.		

© 2021 Nordh M et al. *JAMA Psychiatry.*

eTable 5. Treatment completion, credibility, satisfaction and adherence as well as therapist time.

	Mean (SD)		*t*-value	*p*-value
	**ICBT**	**ISUPPORT**		
Completed online modules (child)				
Post	7.53 (2.60)	7.73 (2.53)		
3MFU	7.86 (2.67)	8.33 (2.63)	0.89	.38
Completed online modules (parents)				
Post	4.63 (0.82)	4.81 (0.56)		
3MFU	4.67 (0.79)	4.85 (0.50)	1.38	.17
Completed video sessions	2.63 (0.77)	2.58 (0.83)	-0.32	.75
Treatment credibility	33.29 (9.04)	28.48 (10.13)	2.51	.01
Treatment satisfaction	24.95 (5.07)	20.61 (5.08)	3.91	<.001
Treatment adherence	22.84 (10.10)	25.85 (10.02)	1.51	.14
Therapist time (min/week)	28.85 (16.79)	23.71 (12.30)	1.77	.08

3MFU=3-month follow-up

© 2021 Nordh M et al. *JAMA Psychiatry.*

eTable 6. Observed means, standard deviations and model-implied unstandardized and standardized mean differences (effect size *d*) of primary and secondary outcomes at post treatment.

	ICBT		ISUPPORT		Unstandardized mean difference		Effect size	
	n	*m (sd)*	*n*	*m (sd)*		95% CI	*d*	95% CI
**CSR**								
Pre	51	5.1 (0.9)	52	4.9 (0.9)				
Post	49	4.27 (1.24)	52	4.62 (1.22)	-0.42	[-0.70, -0.13]	0.45	[0.14, 0.75]
**LSAS-C**								
Pre	51	85.25 (24.54)	52	77.44 (28.45)				
Post	40	66.25 (26.40)	44	76.11 (28.77)	-11.06	[-17.45, -4.66]	0.43	[0.18, 0.68]
**LSAS-P**								
Pre	51	96.22 (21.87)	52	86.02 (26.68)				
Post	45	74.09 (30.01)	51	81.69 (35.14)	-13.79	[-20.39, -7.19]	0.56	[0.29, 0.83]
**RCADS-C-dep**								
Pre	51	4.35 (3.07)	52	3.92 (2.71)				
Post	40	3.05 (2.79)	44	3.56 (3.35)	-0.92	[-1.71, -0.13]	0.32	[0.05, 0.59]
**RCADS-P**								
Pre	51	45.96 (17.2)	52	39.54 (14.89)				
Post	45	32.82 (17.16)	51	39.65 (20.10)	-8.84	[-13.34, -4.34]	0.52	[0.26, 0.79]
**CGAS**								
Pre	51	54.51 (7.31)	52	56.96 (9.47)				
Post	49	58.22 (9.17)	50	57.50 (9.29)	2.17	[0.18, 4.17]	0.26	[0.02, 0.50]
**WSAS-P**								
Pre	51	14.65 (7.14)	52	13.04 (6.96)				
Post	45	11.71 (8.34)	51	11.41 (7.66)	-2.26	[-4.17, -0.36]	0.32	[0.05, 0.60]
**CHU9D**								
Pre	51	12.69 (6.25)	52	12.71 (6.42)				
Post	40	9.03 (5.91)	44	10.67 (7.25)	-0.91	[-2.49, 0.66]	0.14	[0.10, 0.38]

© 2021 Nordh M et al. *JAMA Psychiatry.*

eTable 7. Primary and secondary outcomes for participants receiving ICBT 3-months after ISUPPORT. Analysis includes complete cases.

Measures	Pre	Post	3MFU	*t*-value*	*p*-value*
**CSR**					
n	30	27	27		
M(SD)	4.80 (0.96)	4.15 (1.26)	4.12 (1.56)	2.94	.007
**LSAS-C**					
n	29	20	19		
M(SD)	74.83 (24.83)	67.90 (26.77)	63.53 (29.28)	1.38	.19
**LSAS-P**					
n	29	22	23		
M(SD)	81.77 (31.13)	83.45 (29.56)	71.91 (28.52)	1.93	.067
**RCADS-C-dep**					
n	29	19	18		
M(SD)	3.66 (2.70)	2.84 (2.36)	3.11 (2.67)	1.60	.13
**RCADS-P**					
n	29	22	23		
M(SD)	36.76 (17.57)	34.59 (17.26)	32.78 (17.22)	.82	.42
**CGAS**					
n	30	27	27		
M(SD)	58.13 (7.00)	60.74 (7.60)	60.85 (9.53)	-1.80	.084
**WSAS-**P					
n	30	23	23		
M(SD)	11.53 (6.90)	11.65 (6.64)	10.43 (7.71)	.32	.75
**CHU9D**					
n	29	19	18		
M(SD)	9.72 (5.66)	8.79 (4.69)	8.56 (6.27)	.73	.48

CSR=Clinician Severity Rating; CGAS=Children's Global Assessment Scale; LSAS-C/P=Liebowitz Social Anxiety Scale-Child/Parent version; RCADS-C-dep=Revised Children's Anxiety and Depression Scale-Child version-Depression; RCADS-P= Revised Children's Anxiety and Depression Scale-Parent version; WSAS-P=Work and Social Adjustment Scale-Parent version; CHU9D=Child Health Utility 9D. *Note:* Pre=3 month-follow up after ISUPPORT.*=pre-FU3.

© 2021 Nordh M et al. *JAMA Psychiatry.*

eTable 8. Unit costs and sources.

Type of cost	Resource	Cost (Euro)	Source of cost
Healthcare	General practitioner	357.82 € / visit	Region Stockholm
	Nurse	242.63 € / visit	Region Stockholm
	Social worker	260.80 € / visit	Region Stockholm
	Physical therapist	157.67 € / visit	Region Stockholm
	Specialist medical doctor	404.70 € / visit	Region Stockholm
	Psychologist	351.79 € / visit	Region Stockholm
	Speech therapist	242.63 € / visit	Region Stockholm
	Dietician	278.98 € / visit	Region Stockholm
	Alternative medicine	66.59 € / visit	Region Stockholm
Support	Study help	42.10 € / hour	Own benchmark estimate
	Support of friends & family	13.80 € / hour	Average hourly salary minus tax (30%)
	Personal assistance	27.84 € / hour	Municipalities and regions in Sweden
	Support family	76.25 € / day	Municipality and regions in Sweden
	Loss of parent's leisure time	13.80 € / hour	Average hourly salary minus tax (30%)
Medicines	Medicines (pills / dosages)	Market prices	Dental and Pharmaceutical Benefits Agency, TLV
Supplements	Dietary supplements (pills / dosages)	Market prices	Pharmacy price lists
Absenteeism	Absence from work (parents)	157.86 € / day	Average daily salary in Sweden, Statistics Sweden
	Absence from school (child)	58.85 € / day	Cost per school day, National Agency for Education
Presenteeism	In school when feeling ill (child)	58.85 € / % of day	Cost per school day, National Agency for Education

eTable 9. Resource use frequencies.

	ICBT		ISUPPORT	
	M	***sd***	***M***	***sd***
General practitioner (visits)	0.41	0.69	0.53	1.08
Specialist medical doctor (visits)	0.63	1.24	0.53	1.08
Nurse (visits)	0.26	0.57	0.43	1.11
Social worker (visits)	0.16	0.52	1.99	7.20
Psychologist (visits)	0.85	1.86	0.98	2.25
Speech therapist (visits)	0.02	0.15	0.00	0.00
Dietician (visits)	0.09	0.46	0.04	0.21
Physical therapist (visits)	0.59	2.77	0.11	0.38
Alternative medicine (visits)	0.13	0.54	0.29	1.66
Study help (hours)	2.79	8.37	3.49	12.70
Personal assistance (hours)	0.00	0.00	0.00	0.00
Support family (days)	0.00	0.00	0.00	0.00
Parents' leisure time (hours)	29.78	82.03	31.03	75.03
Medicines (pills/dosages)	36.68	75.60	44.81	99.43
Dietary supplements (pills/dosages)	44.16	87.03	17.12	44.63
Absence from work (days)	1.22	2.94	1.38	5.43
Absence from school (days)	14.88	24.66	10.53	17.46
Specialist teacher (hours)	3.69	17.44	1.98	9.79
In school when feeling ill (days)	8.69	15.72	10.43	23.25

© 2021 Nordh M et al. *JAMA Psychiatry.*

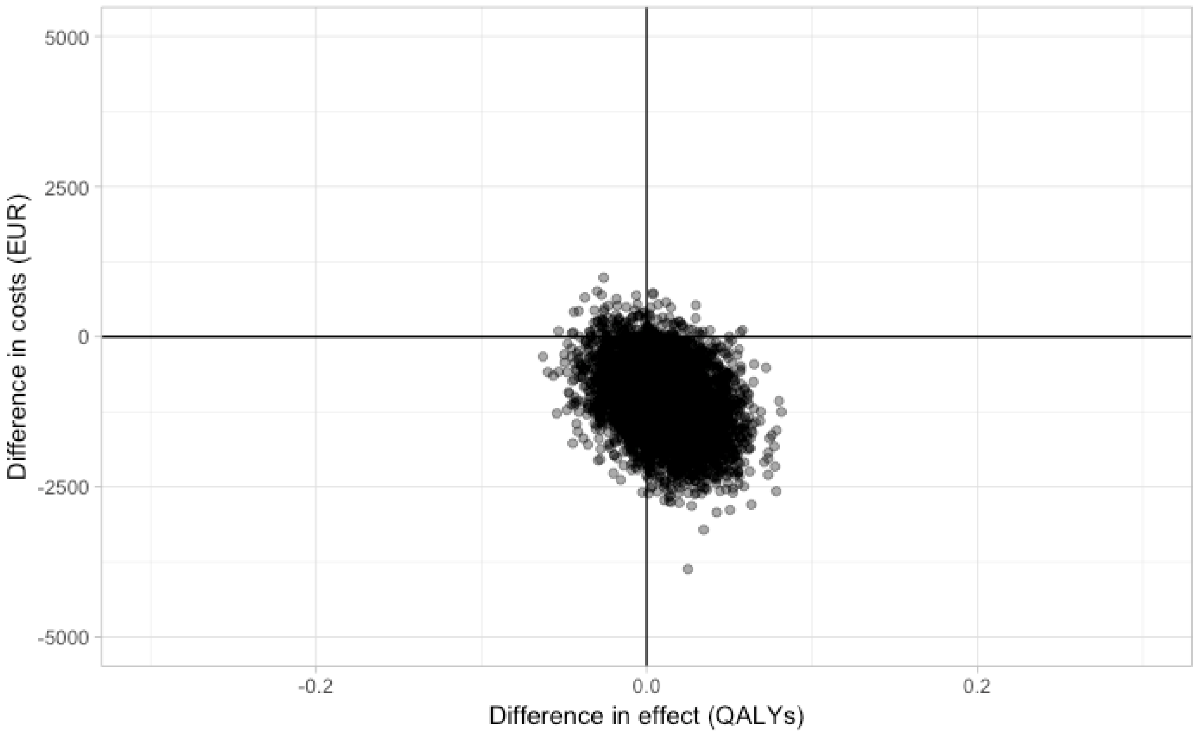

eFigure 1: Cost-effectiveness plane regarding societal cost and QALYs (quality adjusted life years).

© 2021 Nordh M et al. *JAMA Psychiatry.*

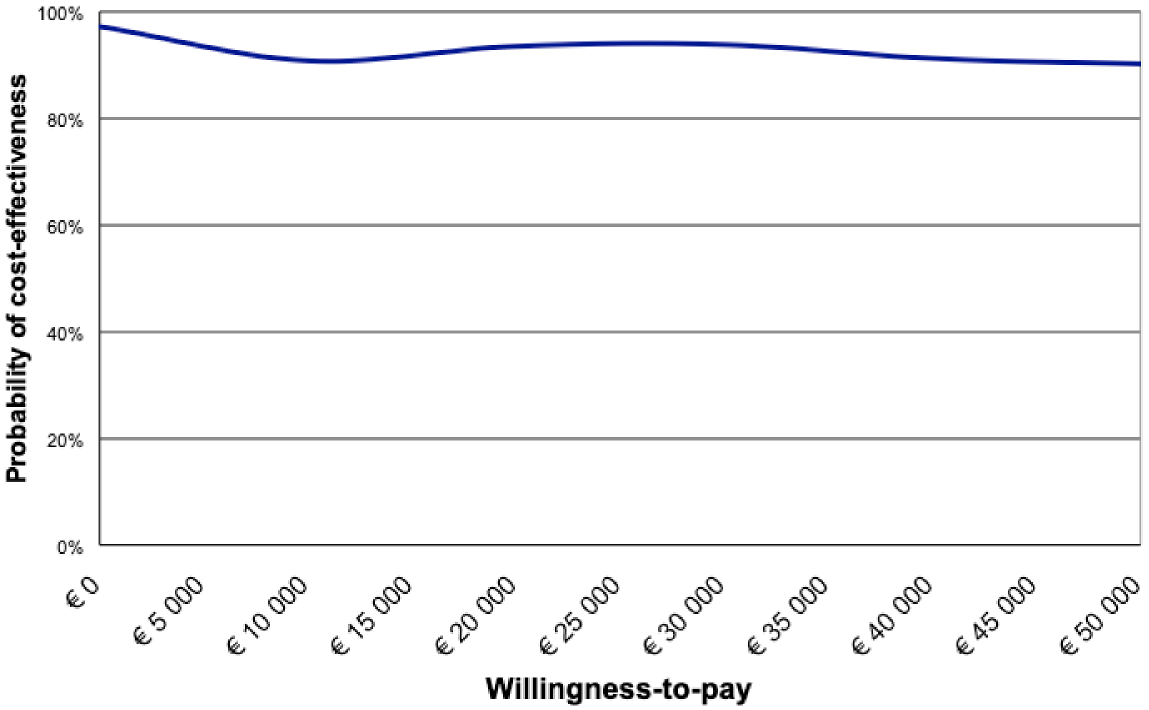

eFigure 2: Willingness-to-pay graph depicting the probability of ICBT to be cost-effective compared to ISUPPORT. Societal perspective (total costs and diagnostic status).

© 2021 Nordh M et al. *JAMA Psychiatry.*

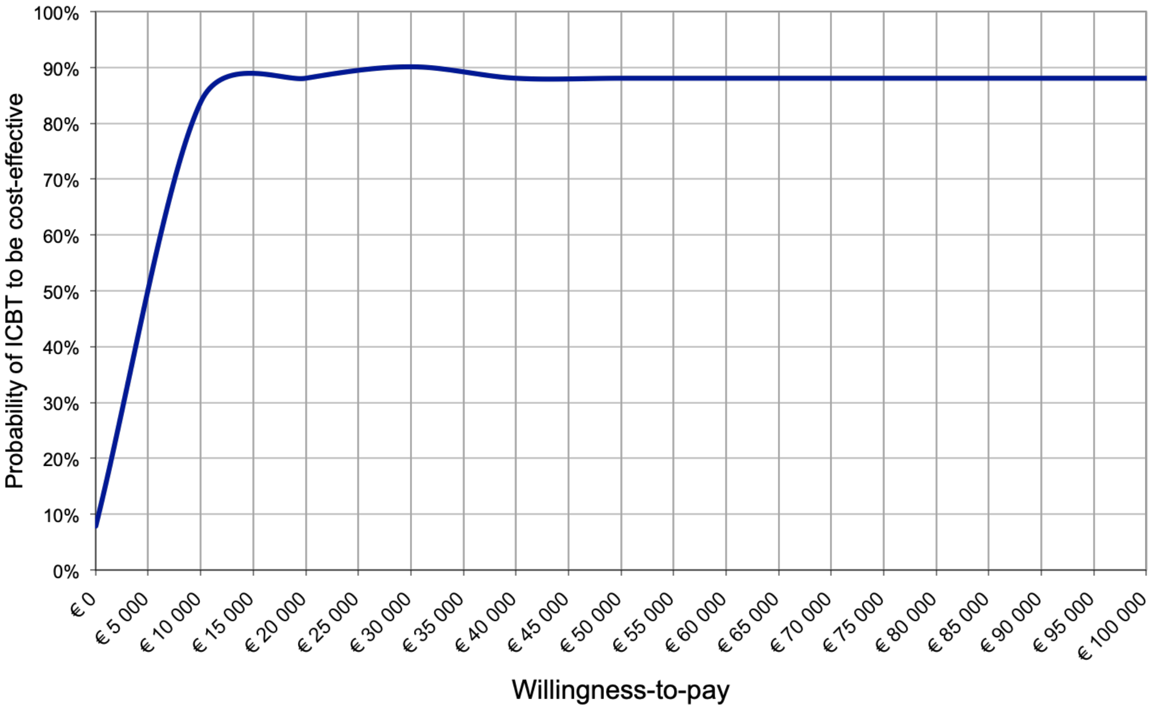

eFigure 3: Willingness-to-pay graph depicting the probability of ICBT to be cost-effective compared to ISUPPORT. Health care provider perspective (treatment costs and diagnostic status).

© 2021 Nordh M et al. *JAMA Psychiatry.*

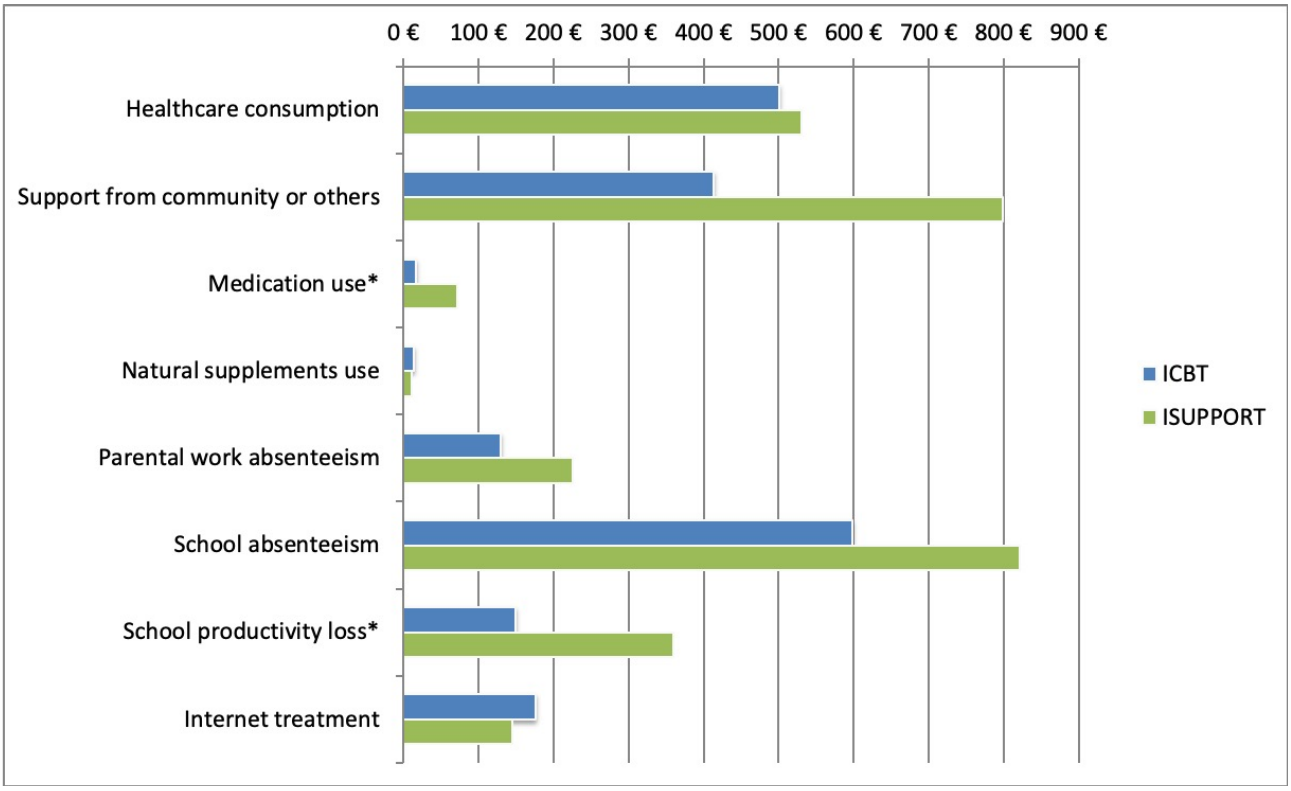

eFigure 4. Sub-total cost differences between ICBT and ISUPPORT during the period from baseline to 3-month follow-up. *p < 0.05.

© 2021 Nordh M et al. *JAMA Psychiatry.*
