# Supplementary material for: Therapist-Guided Internet-Delivered Cognitive Behavioral Therapy vs Internet-Delivered Supportive Therapy for Children and Adolescents With Social Anxiety Disorder: A Randomized Clinical Trial
Source: JAMA Psychiatry. 2021 May 12;78(7):1–9. doi: 10.1001/jamapsychiatry.2021.0469 (PMC8117054; doi:10.1001/jamapsychiatry.2021.0469)
Supplement: Supplement 3. — Data Sharing Statement. [file jamapsychiatry-e210469-s003.pdf]

Data Sharing Statement

Nordh. Therapist-Guided Internet-Delivered Cognitive Behavioral Therapy vs Internet-Delivered Supportive Therapy for Children and Adolescents With Social Anxiety Disorder. *JAMA Psychiatry*. Published May 12, 2021. doi:10.1001/jamapsychiatry.2021.0469

Data
Data available: No

Additional Information
Explanation for why data not available: The datasets generated and analyzed during the current study are not publicly available due to national (Swedish) and EU legislation, but are available from the corresponding author upon reasonable request.
